# *Sinorhizobium meliloti* YbeY is a zinc-dependent single-strand specific endoribonuclease that plays an important role in 16S ribosomal RNA processing

**DOI:** 10.1093/nar/gkz1095

**Published:** 2019-11-28

**Authors:** Vignesh M P Babu, Siva Sankari, James A Budnick, Clayton C Caswell, Graham C Walker

**Affiliations:** 1 Department of Biology, Massachusetts Institute of Technology, Cambridge, MA 02139, USA; 2 Department of Biomedical Sciences and Pathobiology, VA-MD College of Veterinary Medicine, Virginia Tech, Blacksburg, VA, USA; 3 Department of Microbiology and Molecular Genetics, School of Medicine, University of Pittsburgh, PA, USA

## Abstract

Single-strand specific endoribonuclease YbeY has been shown to play an important role in the processing of the 3′ end of the 16S rRNA in *Escherichia coli*. Lack of YbeY results in the accumulation of the 17S rRNA precursor. In contrast to a previous report, we show that *Sinorhizobium meliloti* YbeY exhibits endoribonuclease activity on single-stranded RNA substrate but not on the double-stranded substrate. This study also identifies the previously unknown metal ion involved in YbeY function to be Zn^2+^ and shows that the activity of YbeY is enhanced when the occupancy of zinc is increased. We have identified a pre-16S rRNA precursor that accumulates in the *S. meliloti ΔybeY* strain. We also show that *ΔybeY* mutant of *Brucella abortus*, a mammalian pathogen, also accumulates a similar pre-16S rRNA. The pre-16S species is longer in alpha-proteobacteria than in gamma-proteobacteria. We demonstrate that the YbeY from *E. coli* and *S. meliloti* can reciprocally complement the rRNA processing defect in a *ΔybeY* mutant of the other organism. These results establish YbeY as a zinc-dependent single-strand specific endoribonuclease that functions in 16S rRNA processing in both alpha- and gamma-proteobacteria.

## INTRODUCTION

*ybeY* is one of the required genes in the postulated minimal bacterial gene set and encodes a member of the UPF0054 protein family (1). The first phenotypic characterization of any *ybeY* mutant was described in an earlier study, which showed that the *ybeY* gene (SMc01113) is critically required for *Sinorhizobium meliloti* symbiosis with alfalfa (*Medicago sativa*) plants (2). In addition to being symbiotically defective, loss of YbeY also rendered the *S. meliloti* cells sensitive to various stresses. *Escherichia coli* has been since utilized as the model system to extensively investigate the molecular mechanisms of YbeY function both *in vivo* and *in vitro*. In *E. coli*, YbeY is part of the heat shock response genes and is essential for growth at higher temperatures (3,4). These studies showed that loss of YbeY results in the accumulation of 17S rRNA, the precursor of 16S rRNA (4) while after a shift to elevated temperatures, no mature 16S was detected in the strain lacking YbeY. These observations indicate that the protein plays a critical role in 16S rRNA processing.

Prokaryotic 70S ribosomes contain the 23S and the 5S ribosomal RNAs (rRNA) in the 50S subunit and the 16S rRNA in the 30S subunit (5,6). These ribosomal RNAs originate from a single primary transcript which is processed initially by RNase III cleavage events that happen at the stem regions of the RNA secondary structures (7,8). In *E. coli*, maturation of the 5′ end of the 16S rRNA involves endoribonucleolytic cleavages by RNase E followed by RNase G (9,10). In addition to YbeY, four exoribonucleases (RNase R, RNase PH, PNPase, RNase II) have been implicated in the maturation of the 3′ end of the 16S rRNA (11). Although these studies have demonstrated a pathway to achieve complete 16S rRNA maturation at both ends, additional proteins have been shown to be important for this process. For example, loss of YqgF results in accumulation of 16S precursors *in vivo* and purified YqgF is capable of maturing these precursors *in vitro* indicating that this protein acts directly on 16S rRNA (12).

YbeY has been shown to be a single-strand specific endoribonuclease with relatively little sequence specificity (13–15). Although YbeY can degrade total rRNA substrates, it is not capable of degrading a double-stranded RNA substrate (13). Mutations affecting YbeY’s presumed catalytic site results in the accumulation of 17S precursor, observations consistent with at least one of YbeY’s roles in the processing of the 16S rRNA 3′-end requiring its single-strand endoribonuclease activity. Despite the very limited intrinsic specificity of its single-strand endoribonuclease activity, several pieces of evidence suggest that YbeY can be guided to play its highly specific roles through interactions with other proteins. Using a bacterial two-hybrid system, YbeY has been shown to interact with ribosome-related proteins such as Era, Der and S11, as well as with SpoT and YbeZ (16). S11 and Era interactions could guide YbeY to the pre-16S substrate. Furthermore, YbeY interaction with Era has been suggested to play an important role in stabilizing the 3′ end of the 16S precursor rRNA for processing (17). YbeY together with RNase R has been proposed to act in 70S ribosome quality control removing defective ribosomes (13). YbeY also plays a role in sRNA regulation in bacteria and interestingly shares some conserved features with Argonaute proteins (18,19).

Structures of the YbeY homologs from *Aquifex aeolicus*, *Thermotoga maritima* and *E. coli* have been solved using X-ray crystallography or NMR (20–22). YbeY proteins have a single channel and a histidine triad that coordinates the presumed catalytic metal ion. The physiological roles of YbeY have also been studied in numerous other organisms. In *Vibrio cholerae*, YbeY has been shown to be important for 16S rRNA processing and pathogenicity (15). In enterohemorrhagic *E. coli*, ribosome maturation by YbeY stabilizes a type 3 secretion system transcript required for virulence (23), while in *Yersinia enterocolitica* loss of YbeY results in the growth defects, 16S processing defects and decreased infectivity (24). In *Corynebacterium glutamicum*, YbeY is implicated in the maturation of 4.5S rRNA (25). In *Agrobacterium tumeficiens*, loss of YbeY has been shown to result in accumulation of 16S rRNA precursors (26), while in *Brucella abortus* loss of YbeY resulted in sensitivities to various stresses and significantly reduced the ability to grow in macrophages (27). *ybeY* is also identified to be one of the genes required for longevity under carbon starvation conditions in *Rhodopseudomonas palustris*, a phototrophic alpha-proteobacterium (28). In *Arabidopsis thaliana*, YbeY is thought to localize to the chloroplasts and plays an important role in the maturation of chloroplast rRNAs (29). The YbeY homolog in humans (*c21orf57* gene product) has been demonstrated to be able to partially suppress the defects of an *E. coli* strain lacking YbeY (30).

Recently, Saramago *et al.* (31) studied the *in vivo* and *in vitro* characteristics of *S. meliloti* YbeY (SmYbeY) and reported several results that differed strikingly from other studies of this highly conserved protein. Firstly, they reported that SmYbeY is an endoribonuclease with unprecedented catalytic features, concluding that it has a double-strand endoribonuclease activity as well as a single-strand endoribonuclease activity. Secondly, they reported that SmYbeY required the addition of Mg^2+^ or Mn^2+^ for its activity. YbeY belongs to the UPF0054 family, whose members are predicted to coordinate a metal ion by three histidine residues in its active site similar to many metallo-proteinases (20). These metallo-proteinases are known to bind and utilize zinc as a cofactor (32). One prior study incubated purified *Thermotoga maritima* YbeY with zinc and reported its ability to bind zinc in a 1:1 ratio (21). Thirdly, Saramago *et al.* did not observe any role for SmYbeY in the rRNA processing in *S. meliloti*. Ribosomal RNA processing in the alpha-proteobacterium *S. meliloti* has some striking differences from gamma-proteobacteria. Many of the processing steps in *S. meliloti* are predicted and have not been experimentally verified. Unlike *E. coli* but like many alpha-proteobacteria, the 23S rRNA of *S. meliloti* undergoes further cleavage to yield the 2.6 kb rRNA and a 5.8S like rRNA (33). RNase J is present in *S. meliloti* but not in *E. coli* and is implicated in the processing of both 16S and 23S rRNAs (34). However, given that the role of YbeY in rRNA processing observed in a wide range of organisms, it would be unexpected for YbeY not to participate in rRNA processing in *S. meliloti* as well.

In this study, we have characterized the biochemical activity of SmYbeY and revisited the *in vivo* role of SmYbeY in *S. meliloti*. We show that the purified SmYbeY protein exhibits only a single-strand specific endoribonuclease activity and does not act on a double-stranded substrate under normal conditions. We also show that YbeY is a zinc-containing enzyme and increased occupancy of zinc in the protein sample enhances its activity. As in other bacteria, loss of YbeY in *S. meliloti* results in growth defects and impaired 70S ribosome assembly and also leads to the accumulation of a novel pre-16S species which is longer than its *E. coli* counterpart 17S rRNA. We predict that the pre-16S species in alpha-proteobacteria would be longer compared to that of gamma-proteobacteria. However, unlike in *E. coli*, heat shock has only a minor effect on rRNA processing in *S. meliloti*.

## MATERIALS AND METHODS

### Purification of YbeY

Wild type SmYbeY and SmYbeY R69A were cloned into pET28a vector using NdeISmYbeYFor and BamHISmYbeYRev with a C-terminal Strep-tag II. The plasmid is then transformed into a modified strain (BL21VB1), a derivative of *E*. *coli* strain BL21(DE3) strain that lacks both the *rna* and *pnp* genes and also contains the pRARE2 plasmid, which carries seven rare codon tRNA genes. Cells were grown to mid-exponential phase at 37°C in LB media and expression of YbeY was induced with 1mM IPTG for 4 h. Cells were collected using centrifugation at 8000g for 30 min and re-suspended in a lysis buffer (25 mM Tris–HCl pH 7.5; 200 mM Potassium acetate; 1 mM DTT; cOmplete Mini EDTA free protease inhibitor (Roche)). Cells were then lysed mechanically using a French press. Cell lysates were separated by centrifugation at 10 000g for 15 min and passed through a 0.45 μm filter. Proteins were purified using the Strep-tactin affinity purification system (IBA Life Sciences) according to manufacturer's instructions except EDTA was omitted from all the buffers. Eluted protein was then loaded onto a size exclusion column (GE Hiload 16/60 Superdex 75 pg) equilibrated with Buffer B (25 mM Tris–HCl pH 7.5; 200 mM potassium acetate; 1 mM DTT). The purity of protein preparations was analyzed on a SDS-PAGE gel (Supplementary Figure S1A) followed by a mass spectrometry. No other RNases were identified to be present in the protein preparations by this analysis. For buffer exchange into metal-free water, purified SmYbeY was loaded onto the HiPrep™ 26/10 desalting column (GE Healthcare) that was equilibrated with metal-free water (VWR Aristar Ultra).

### RNA degradation assay

Single-stranded RNAs was labeled at 5′ end using T4 polynucleotide kinase and ATP(γ^32^P). Template were mixed with YbeY in activity buffer (25 mM Tris–HCl pH 7.5; 200 mM potassium acetate; 1 mM DTT; 10% glycerol) at the mentioned concentrations and incubated for 2 h. 2× loading dye (25% of 20% Ficoll, 50% urea, 0.05% Bromophenol blue and 0.05% xylene xylol) was added to the sample and was run in 20% polyacrylamide gel with 8 M urea. The image was developed using either phosphor screen (Amersham) and visualized in the phosphor imaging program Typhoon FLA 9500 or using X-ray film. Double-strand RNAs (dsRNA) were made by hybridizing ssRNA_1 and ssRNA_1Comp for 30mer dsRNA, 39merRNA and 39merCompRNA for 39mer dsRNA, 20merRNA and 20merCompRNA for 39mer dsRNA using the hybridization protocol as described in (31). For sequence details of the RNAs used, refer to Table 1.

**Table 1.** Strains, plasmids and oligonucleotides used in this study

Bacterial strains		
Strain name	Relevant characteristics	Source
SM1021	*S. meliloti* wild type *ybeY+*	Laboratory stock
SM1021 *SmΔybeY*	SM1021 *SMc01113::mTn5* Neo^R^	(2)
SM2B3001	*S. meliloti* wild type *ybeY+*	Dr Jimenez-Zurdo
SM2B3001 *SmΔybeY*	*ΔybeY* derivative of SM2B3001	Dr Jimenez-Zurdo
MC4100	*E. coli* wild type *ybeY+*	Laboratory stock
*EcΔybeY*	*ΔybeY* derivative of MC4100	(4)
2308	*B. abortus* wild type *ybeY+*	(27)
*BaΔybeY*	2308 *ΔybeY*	(27)
BL21VB1	BL21(DE3) *Δpnp Δrna* pRARE2	This work
**Plasmids**		
pRF771	Empty vector; tet^R^	Laboratory Stock
pRF-SmYbeY	pRF771 with *S. meliloti ybeY* gene cloned between PstI and BamHI sites	This work
pWSK29	Empty vector; pSC101 origin; Amp^R^	Laboratory Stock
pWSK-SmYbeY	pWSK29 with *S. meliloti ybeY* gene cloned between PstI and BamHI sites	This work
pRF-EcYbeY	pRF771 with *E. coli ybeY* gene cloned between PstI and BamHI sites	This work
pET28a	Expression vector; T7 promoter; Kan^R^	Laboratory Stock
pET28a-SmYbeY	pET28a with *S. meliloti ybeY* gene cloned between NcoI and BamHI sites	This work
**Oligonucleotides**		
ssRNA_1	GGUUGGAUCACCUCCUUACCUUAAAGAAGC	Eurofins genomics
ssRNA_2	CCCGACACCAACCACUAAAAAAAAAAAAAA	
ssRNA_1Comp	GCUUCUUUAAGGUAAGGAGGUGAUCCAACC	
39merRNA	GGCUGGAUCACCUCCUUUCUAAGGAAGCUGUGGAAUUGG	
39merCompRNA	CCAAUUCCACAGCUUCCUUAGAAAGGAGGUGAUCCAGCC	
20merRNA	GGUUGGAUCACCUCCUUACC	
20merCompRNA	GGUAAGGAGGUGAUCCAACC	
16S probe	AACTGCCCGACGGCTAACATTCATCGTTTACGGCGTGGACTACCAGG	
23S probe	CGAATATTAACGTGGTTCCCATCGACTACGCGTGTCCGCCTCGTCTTAGG	
PstIybeYFwd	ATTCTGCAGGAAACGAGAGTGCCGTCCCCCATGACGGCATTGGACATTCAG	
BamHIybeYRev	ATTGGATCCTTAATGCGGGGGTTGGTCC	
PstIEcybeYFwd	ATTCTGCAGGAAACGAGAGTGCCGTCCCCCATGAGTCAGGTGATCCTCGA	
BamHIEcybeYRev	ATTGGATCCTTATTCTTTCTCGGCAATGTACG	
NcoISmybeYFor	TGCTGCCATGGGTACGGCATTGGACATTCAGAT	
BamHISmybeYRev	TGCTGGGATCCTTATTATTTTTCGAACTGCGGGTGGCTCCAAGCGCTATGCGGGGGTTGGTCCCCGTAGG	
qrpoBFor	TGACGAGATCGACGAGAAGA	
qrpoBRev	TAGGCGCCGATATTGATGTG	
qybeYFor	AACTGTCGCTGGTCTTCAC	
qybeYRev	GCGGGAAAGGACAGAACAT	
16S_SSPfwd	GGTGAAGTCGTAACAAGGTAG	
16S_SSPRev	GCCAGGATCAAACTCTCAAG	

### ICP-MS

The protein sample was serially diluted in the activity Buffer or metal-free water. 200 μl of each sample was mixed with 2 ml of 10% HNO_3_ and the samples were injected into the ICP-MS (Agilent 7900) and metal content was measured in He mode. A mixture of Mg, Mn, Ni, Co and Zn (1 mM each) was prepared and serially diluted up to 0.1 μM. These standards were analyzed and the metal counts were plotted against concentration as standard curves. The actual metal content of the samples was calculated by fitting the signal with the standard curve for each metal.

### Bacterial strains and growth conditions

*S. meliloti* strains SM1021 and SM2B3001 were routinely grown in LB supplemented with 2.5 mM CaCl_2_ and 2.5 mM MgSO_4_ (LBMC) in the presence of 200 μg/ml Streptomycin at 30°C. When mentioned, *S. meliloti* were grown in minimal media (MM) as described previously (31). *Escherichia coli* strains were routinely grown in LB medium at 37°C. When required tetracycline (5 μg/ml) or ampicillin (150 μg/ml) were added to the growth media. Strains, plasmids and primers used in this study and their sources are listed in the Table 1.

### Construction of plasmids and complementation strains

The YbeY gene was amplified from the genomic DNA of *S. meliloti* 1021 using the primers PstIybeYFwd and BamHIybeYRev and cloned into vector pWSK29 to make pWSK-SmYbeY. This *smybeY* gene was then sub-cloned into the vector pRF771 to make pRF-SmYbeY. EcYbeY from the genomic DNA of *E. coli* (MC4100) was amplified using the primers PstIecybeY Fwd and BamHIecybeYRev and cloned into pRF771 to make pRF-EcYbeY. These plasmids were then transformed into *S. meliloti ΔybeY* mutant using tri-parental mating with the helper strain *E. coli* MT616 and successful complemented strains were selected on LBMC plates containing Streptomycin and Tetracycline.

### Polysome analysis

Polysome analysis was performed as described previously (30) with some modifications. Briefly, cells were resuspended in Buffer A (20 mM HEPES pH 7.5, 5 mM β-mercaptoethanol, 10 mM MgCl_2_, 50 mM NH_4_Cl, 0.1 mM PMSF) and homogenized by passing through a French press. After clarification by centrifugation, the supernatant was loaded to a 10–40% sucrose gradient and ultracentrifugation was performed at 38 000g for 4 h. 300 μl fractions were collected and *A*_260_ was measured for each fraction.

### Northern blot

Total RNA from the indicated strains were subjected to Agarose/Synergel (1.6%) electrophoresis and transferred to Hybond N+ membrane overnight through capillary electrophoresis in 10× SSC buffer. The membrane was then UV cross-linked (1200 J) using a Stratagene UV crosslinker and was pre-hybridized with Ultrahyb oligo Hybridization buffer (Invitrogen) for 30 min. 0.2 pmol of either 16S or 23S probe which were 5′ labeled using T4 Polynucleotide Kinase (NEB), was then added and allowed to hybridize overnight at 65°C. After washing with 2× SSC and 0.5% SDS, the image was developed in phosphor screen (Amersham) and visualized using the phosphor-imaging software of Typhoon FLA 9500.

### RNA extraction and rRNA profiling

Mid-log phase cultures were collected, and total RNA was extracted using Trizol (Thermo Fisher Scientific) method. Qiagen RNAeasy kit was used to purify the RNA. Total RNA was mixed with 2× RNA loading dye (NEB)and heated for 5 min at 65°C and subsequently run in a 1.6% Agarose/Synergel as described previously (17).

### *Brucella abortus* growth and RNA extraction

The strains used in this experiment were *Brucella abortus* 2308 and a previously described strain containing an unmarked in-frame isogenic deletion of *ybeY*, *B. abortus* 2308::*ΔybeY* (27). The strains were routinely grown on Schaedler blood agar (SBA), which is composed of Schaedler agar (VWR International LLC, Radnor, PA) containing 5% defibrinated bovine blood (Quad Five, Ryegate, MT) or in brucella broth (VWR). For RNA collection, cultures of the *B. abortus* strains were grown to the late exponential phase of growth in brucella broth with shaking (200 rpm) at 37°C. An equal volume of 1:1 ethanol–acetone was added to the cultures, and the mixture was stored at −80°C. RNA was isolated from *B. abortus* cultures as described previously (35). Briefly, cultures were thawed, and the cells were collected by centrifugation at 18 000g for 5 min at room temperature. RNA was extracted from the cells using the TRIzol reagent (Life Technologies Corporation, Carlsbad, CA, USA), followed by ethanol precipitation of the RNA.

### 5′ RACE and 3′ RACE protocol

Total RNA was isolated by using the Trizol method. For 5′ RACE, the first-strand cDNA was synthesized using the primer 16S_SSPrev (Table 1) specific to the 16S rRNA. RNAse H (NEB) was used to remove the RNA from the resulting RNA–DNA hybrid. Terminal transferase (NEB) was used to add a poly-A tail to the 5′ end of DNA. The resulting strand of DNA was used as a template and poly-T adapter primer was used to synthesize the other strand of DNA. This product was amplified using adapter fwd and SSP rev and sequenced using SSP rev to determine the 5′ end. For 3′RACE, polyA tail was added to the 3′ end of RNA using poly-A polymerase. Adapter poly-T primer was used to synthesize the first strand of DNA. Second-strand was synthesized using the primer 16S_SSPfwd. The product was PCR amplified using SSP for and Adapter Rev and sequenced to find the 3′ end.

### Computational tools

Conservation scores for the amino acids in a protein sequence were obtained using the Consurf tool (http://consurf.tau.ac.il/) (36). Protein structures were visualized and rendered using Pymol 2.0.5 (https://pymol.org). Protein docking with the RNA substrates was performed using Cluspro (https://cluspro.bu.edu) (37–39). Protein structures were obtained from PDB (https://rcsb.org). Homology modeling of SmYbeY was performed using SWISS-MODEL (https://www.swissmodel.expasy.org/) (40). Zinc binding probabilities of the protein was obtained using ZincExplorer (http://systbio.cau.edu.cn/ZincExplorer/) (41).

## RESULTS

### *Sinorhizobium meliloti* YbeY is a single-strand specific endoribonuclease

**Figure 1.**
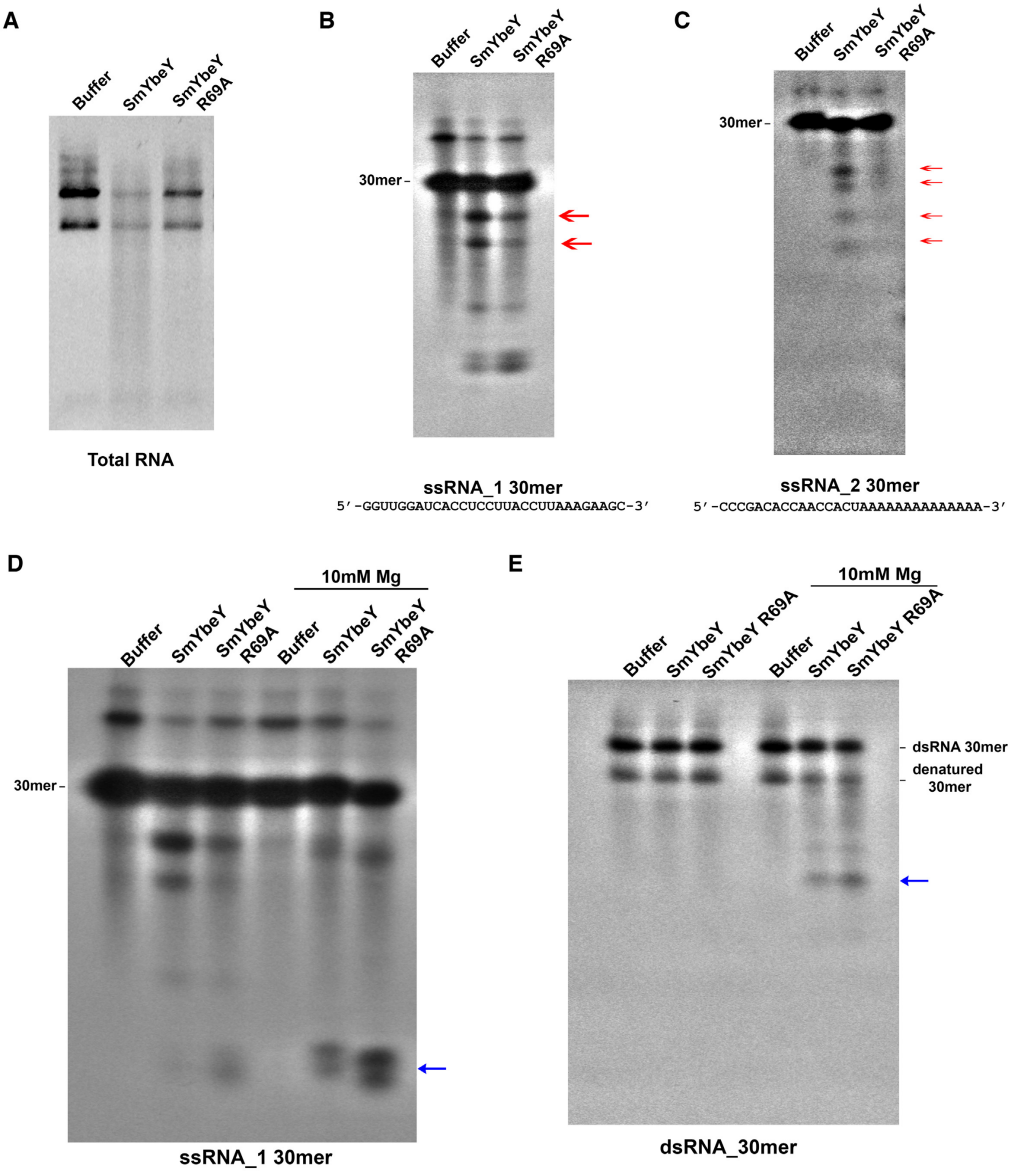
YbeY is a single-strand specific endoribonuclease. (**A**) Total RNA from *S. meliloti* wild type cells were extracted and incubated with activity buffer or 25 μM wild type *S. meliloti* YbeY (SmYbeY) or 25 μM YbeY carrying R69A mutation (SmYbeY R69A). The reaction mixture subjected to electrophoresis in an agarose/synergel mix gel. (**B** and **C**) SmYbeY activity on two different single-stranded RNA substrates. ^32^P labeled single-stranded RNA substrates were incubated for 1 h at 37°C with the activity buffer or 25 μM SmYbeY or 25 μM SmYbeY R69A. The reaction mixtures were run on a 20% polyacrylamide TBE–urea gel. The major degradation products are indicated by red arrows. (**D**) ^32^P labeled single-stranded RNA substrate (ssRNA_1) was used to perform degradation assays similar to panels B and C, either in the presence or absence of 10mM Mg^2+^. Blue arrow indicates the minor degradation products produced in the presence of Mg^2+^. (**E**) Double-stranded RNA substrate labeled with ^32^P on one strand was incubated with activity buffer or 25 μM SmYbeY or 25 μM SmYbeY R69A, either in the presence or absence of 10 mM Mg^2+^. Blue arrow indicates the minor degradation products produced in the presence of Mg^2+^. Panels are representative of at least three independent experiments.

To purify SmYbeY, we constructed a derivative carrying a C-terminal Strep-tag II and expressed it in BL21VB1, a derivative of *E. coli* strain BL21(DE3) strain that lacks both the *rna* and *pnp* genes and also contains the pRARE2 plasmid, which carries seven rare codon tRNA genes (see Materials and Methods). The use of the Strep-tactin affinity purification system (IBA Life sciences) and a size exclusion chromatography step allowed us to avoid a metal affinity purification step that might displace the native metal ion. In addition, EDTA was omitted from all the buffers to avoid displacing the native metal. The SmYbeY protein preparations we obtained displayed RNase activity without the need for the addition of metals and no other RNases were detectable by mass spectrometry. We also purified a mutant SmYbeY R69A in which the highly conserved arginine residue is replaced by an alanine (Supplementary Figure S1B). *Escherichia coli* YbeY carrying the corresponding change (R59A) has been shown to have reduced single-strand endoribonuclease activity compared to the *E. coli* wild type YbeY (13). Similar to *E. coli* YbeY (13), our preparation of SmYbeY was able to degrade total RNA from its native host without the addition of any exogenous metal. As expected, the SmYbeY R69A mutant has reduced degradation activity (Figure 1A).

The SmYbeY we purified is a single-strand endoribonuclease that does not require the addition of exogenous metal ions. It cleaves ssRNA_1, the 30mer single-stranded RNA substrate we have used previously (13), in a fashion that is extremely similar to *E. coli* YbeY and *V. cholerae* YbeY (13,15), yielding at least two distinct major degradation products (Figure 1B). As expected, the SmYbeY R69A mutant has reduced activity compared to the wild type strain on the single-stranded substrate. The distinct degradation pattern we observed is strikingly different from the Mg^2+^-dependent single-strand RNase activity reported by Saramago *et al.* (31). The Mg^2+^-dependent RNase activity in their SmYbeY preparation instead generated a ladder-like series of multiple bands that included a particularly prominent 3-mer when K^+^ was present. In case the strikingly different pattern of degradation observed by Saramago *et al.* (31), was due to their use of a different 30mer single-strand RNA substrate, we examined the ability of our SmYbeY preparation to degrade the same substrate they used (ssRNA_2). However, our SmYbeY preparation again exhibited a pattern of endoribonuclease cleavage that was highly similar to that observed with ssRNA_1, with four distinct cleavage products and no ladder-like pattern (Figure 1C) (31). We note that the concentration of Mg^2+^ in bacterial cytoplasm is *ca*. 1 mM (42), so 10 mM Mg^2+^ is supra-physiological. Nevertheless, we attempted to see if there is a different cleavage pattern in the presence of Mg^2+^. In the presence of 10mM Mg^2+^, the major products produced by SmYbeY were less pronounced and additional shorter cleavage products were produced (Figure 1D). It should be noted that the shorter cleavage products were produced by both SmYbeY and SmYbeY R69A preparations with the latter producing the most. These observations strongly support the explanation that these products are formed due to the presence of a minor co-purifying contaminant that possesses an Mg^2+^-dependent RNase activity. The reduction of the major degradation products in the presence of Mg^2+^ suggests that the native YbeY activity is partially inhibited by Mg^2+^.

We had previously reported that *E. coli* YbeY did not degrade a double-strand RNA substrate (13) and observed that our purified SmYbeY similarly did not display double-strand endoribonuclease activity in the absence of added metals (Figure 1E, first three lanes). In contrast, Saramago *et al.* (31) reported that their preparation of SmYbeY exhibited a remarkable ability to degrade a double-strand RNA substrate in the presence of 10 mM Mg^2+^, with all of the dsRNA substrate consumed within 30 min. However, when we assayed our SmYbeY preparation for double-strand endoribonuclease activity in the presence of 10 mM Mg^2+^-containing buffer as Saramago *et al.* (31), there was only minor degradation in both the wild type and R69A in the presence of 10 mM Mg^2+^ (Figure 1E; last three lanes). The fact that this minor degradation was only observed in the presence of supra-physiological levels of magnesium and the equivalent minor degradation was observed with the R69A mutant suggests that the dsRNA endoribonuclease activity observed is due to some minor contaminating RNase rather than SmYbeY. In order to compare the cleavage patterns with other RNases, we performed the single-strand and double-strand degradation assays using RNase III and RNase R (Supplementary Figure S2). As expected, RNase III did not cleave single-stranded RNA but cleaved double-stranded RNA in an Mg^2+^ dependent manner. The double-strand activity of SmYbeY reported by Saramago *et al.* (31), was robust and resulted in complete degradation of the double-stranded substrate similar to RNase III degradation (Supplementary Figure S2B and C). Moreover, Saramago *et al.* (31) and, Jiménez-Zurdo and Robledo (43) remark that the specific cleavages their SmYbeY preparation causes in the generic highly structured R1.1 RNA, which is typically used to assay RNase III activity, remarkably resemble the cleavages that RNase III causes on the same substrate. This observation suggests a simple alternative explanation for the authors’ observation of double-strand endoribonuclease activity, namely that their SmYbeY preparation was contaminated by small amounts of an *E. coli* RNase, most likely RNase III.

Since the structures of several YbeY orthologs have been solved, we investigated whether it was plausible that a YbeY protein could productively interact with both single and double-stranded RNA. A homology model of SmYbeY protein was generated by SWISS-MODEL using the *E. coli* YbeY as the template (40). Protein–RNA docking analysis was performed with the modeled SmYbeY and sample single and double-stranded RNA structures. Ten best fits each for single and double-stranded RNA on YbeY were obtained (Supplementary Figure S3). Similar to our previous analysis with *E. coli* YbeY (19), single-strand RNA was able to fit within the active site of the YbeY protein, making contacts with the highly conserved residues present within the active site (Supplementary Figure S4). In striking contrast, double-stranded RNA failed to fit within the active site. The major sites of predicted binding for the double-stranded RNA were the unstructured loops on the outer surface containing less conserved residues that are not predicted to co-ordinate with any metal ion required for cleavage. These results suggest that the structure of YbeY makes it unlikely that SmYbeY could interact productively with double-stranded RNA in a manner that would allow it to catalyze an endonucleolytic incision.

### *Sinorhizobium meliloti* YbeY is a zinc dependent enzyme

The metal present in YbeY and other members of the UPF0054 family has been widely speculated to be zinc: (i) prior to the discovery of its single-strand endoribonuclease activity (13) YbeY orthologs were commonly predicted to be zinc-dependent hydrolases (20,21); (ii) metal-free *Thermatoga maritima* YbeY was shown to form an almost 1:1 complex with zinc (21) and (iii) Penhoat *et al.* pointed to the structural similarities between YbeY and the zinc-containing histidine triad matrix metallo-proteinases (MMPs), including discussing how the Met residue located in the β turn just after the catalytic helix could provide the hydrophobic packing previously described for stabilizing the Zn^2+^-binding motif of MMPs (21). Using the tool ZincExplorer, we observe that His126, His130 and His136 residues of SmYbeY have a high probability to bind zinc (Supplementary Figure S5).

**Figure 2.**
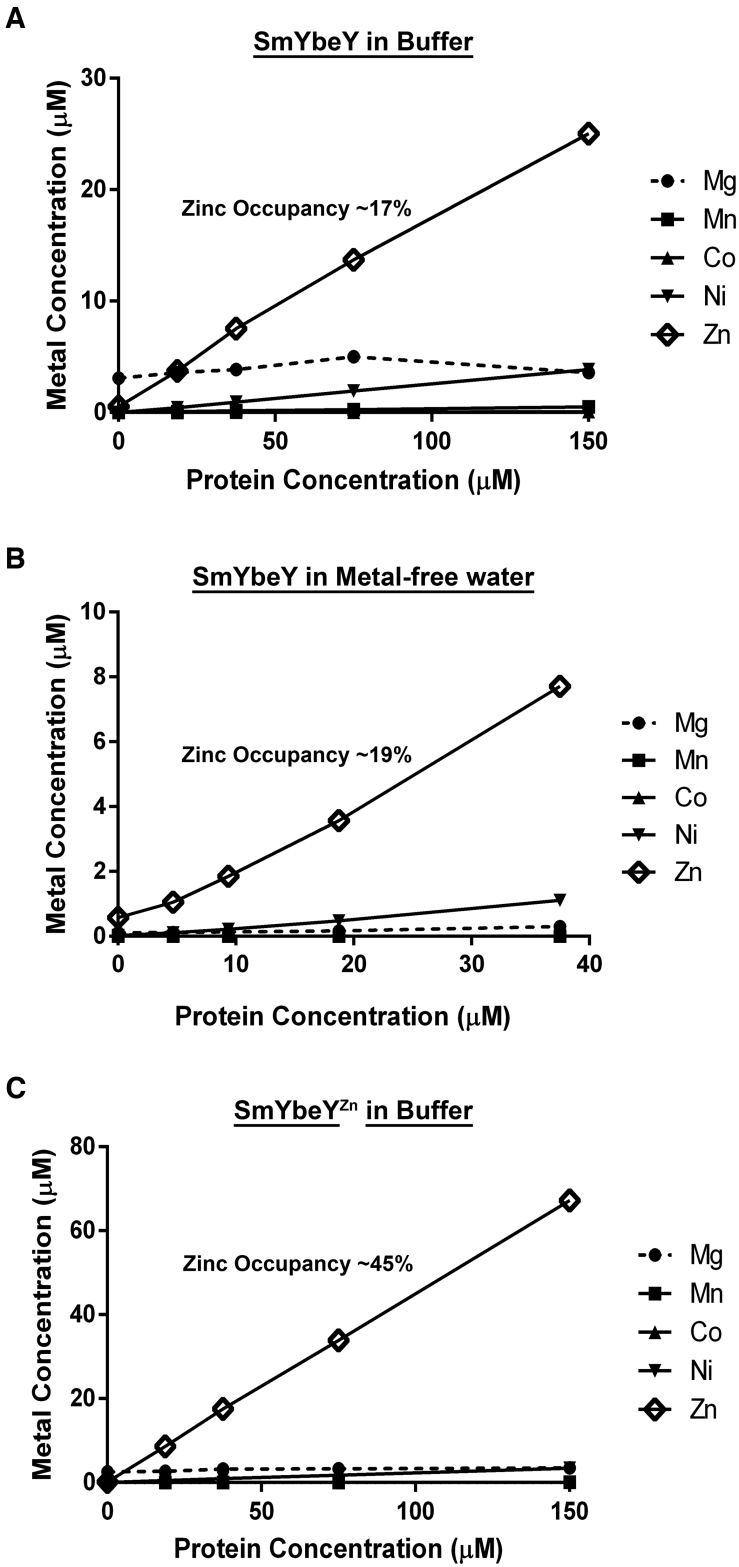
*S. meliloti* YbeY co-ordinates zinc. (**A**) Different concentrations of the SmYbeY protein were prepared in activity buffer and the metal concentrations were calculated for magnesium (Dashed, filled circle), manganese (filled square), cobalt (filled triangle), nickel (inverted triangle) and zinc (open diamond) using ICP-MS. Unlike other metals, the measured concentration of Zn was proportional to the concentration of the protein used. (**B**) YbeY protein was exchanged from Buffer into metal-free water and analyzed using ICP-MS. Zinc was found to be stably associated with the SmYbeY protein. (**C**) SmYbeY was purified from cells supplemented with 0.1 mM Zn (SmYbeY^Zn^) and the metals in the sample were analyzed using ICP-MS. By this method, the zinc occupancy of the protein was further enriched.

To determine which metal is required for SmYbeY RNase activity, we measured the metal content of our protein sample using ICP-MS. Our analysis shows that the concentration of zinc detected increases with the increase in the amount of protein used indicating that YbeY is a zinc-containing protein (Figure 2A). The occupancy of the Zn^2+^ (molar ratio of Zn^2+^ to protein in percentage) in the sample was calculated to be ∼17% whereas the occupancy of nickel was calculated to be ∼2.5%. The occupancies of other metals namely Mg^2+^, Mn^2+^ and Ca^2+^ were < 1%. We then buffer exchanged the protein into metal-free water and subjected it to ICP-MS analysis. Under these conditions, zinc remained stably bound to YbeY (Figure 2B). These results show that the metal normally bound to YbeY is highly likely to be a Zn^2+^ ion and is unlikely to be Mg^2+^, Mn^2+^ and Ca^2+^. We hypothesized that Zn^2+^ occupancy was less than stoichiometric because we were expressing the protein to very high levels in *E. coli* without supplementing the LB medium with Zn^2+^. A previous study successfully showed that properly folded Ada protein can be produced by supplementing the overproduction medium with 0.1mM Zn^2+^ (44). We, therefore, supplemented LB with 0.1 mM Zn^2+^ when 1 mM IPTG is added for inducing protein expression. The Zn^2+^ content of the protein purified by this method (SmYbeY^Zn^) was then analyzed using ICP-MS and zinc occupancy was found to have increased to ∼45% (Figure 2C). This result suggests that the availability of zinc is limited for SmYbeY during overproduction resulting in non-metallated protein so that only some of the SmYbeY molecules in the preparation are catalytically active.

**Figure 3.**
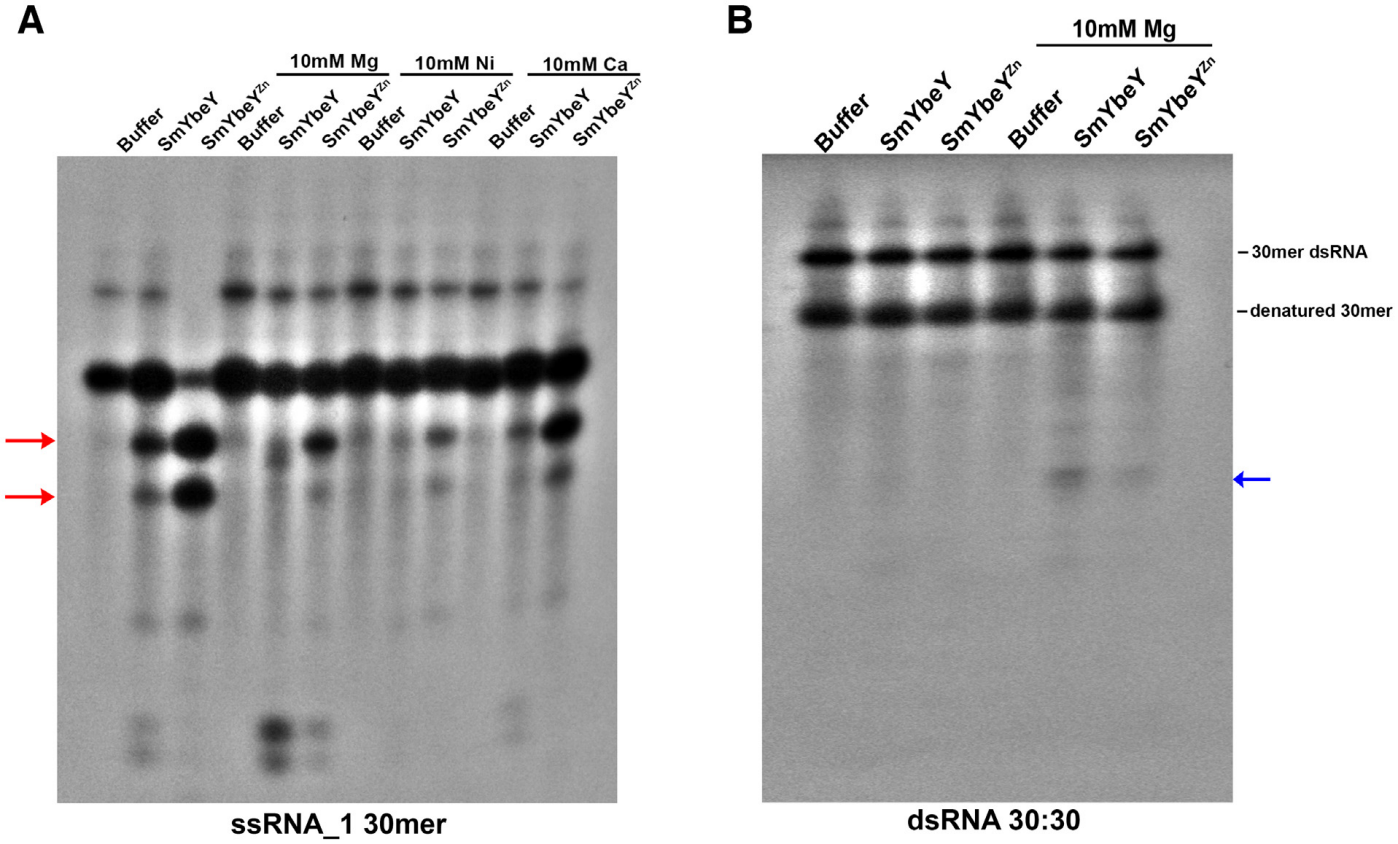
Effect of added metals on SmYbeY activity. (**A**) ^32^P labeled Single-stranded RNA substrate (ssRNA_1) was incubated for 1 h at 37°C with the buffer or 25 μM SmYbeY or 25 μM SmYbeY^Zn^ in the presence or absence of 10 mM Mg^2+^ or 10 mM Ni^2+^ or 10 mM Ca^2+^. The reaction mixture was run on a 20% polyacrylamide TBE-Urea gel. The major degradation products are indicated by red arrows. Increased intensity of the single-strand RNA degradation products is evident when SmYbeY^Zn^ protein was used compared to when the SmYbeY protein was used. (**B**) Double-stranded RNA substrate labeled with ^32^P on one strand was incubated with activity buffer or 25 μM SmYbeY or 25 μM SmYbeY^Zn^, either in the presence or absence of 10 mM Mg^2+^. No degradation of double-strand RNA was observed even when SmYbeY^Zn^ protein was used. Blue arrow indicates the minor degradation products produced only in the presence of Mg^2+^. Panels are representative of at least three independent experiments.

Consistent with this interpretation, we found that the SmYbeY purified from cells grown in zinc-supplemented media (SmYbeY^Zn^) had markedly increased endonucleolytic activity on a single-stranded substrate compared to the SmYbeY purified without zinc supplementation (Figure 3A, first three lanes). We then added excess amounts of Mg^2+^ (10 mM) or Ni^2+^ (10 mM) or Ca^2+^ (10 mM) to the single-strand RNA degradation assay. We find that the addition of excess Mg^2+^ and Ni^2+^ results in the reduction in the formation of the major degradation products whereas addition of Ca^2+^ had only a minor effect (Figure 3A). This result indicates that the presence of the other divalent metals inhibits the endonucleolytic activity of SmYbeY. Using the more highly active SmYbeY^Zn^, we again attempted to detect any double-strand endoribonuclease activity. However, similar to our original observations, SmYbeY^Zn^ also did not show any endoribonucleolytic activity on the double-stranded 30mer RNA substrate (Figure 3B). In fact, the SmYbeY^Zn^ had even less of the Mg^2+^ dependent minor degradation products of the double-stranded RNA observed when SmYbeY is used. This result as well strongly suggests that SmYbeY does not possess a double-strand RNase activity. Our previous report that *E. coli* YbeY does not possess a double-strand RNase activity was performed using a 20mer double-stranded RNA while Saramago *et al.* (31), utilized a 39mer double-stranded RNA for their analysis that showed double-strand activity. In order to rule out the possibility that the length of the substrate contributes to the activity, in addition to the 30mer double-strand, we also performed that degradation assays on 20mer and 39mer double-strand substrates. We were unable to detect a double-strand activity for SmYbeY and SmYbeY^Zn^ on either of these substrates (Supplementary Figure S6).

**Figure 4.**
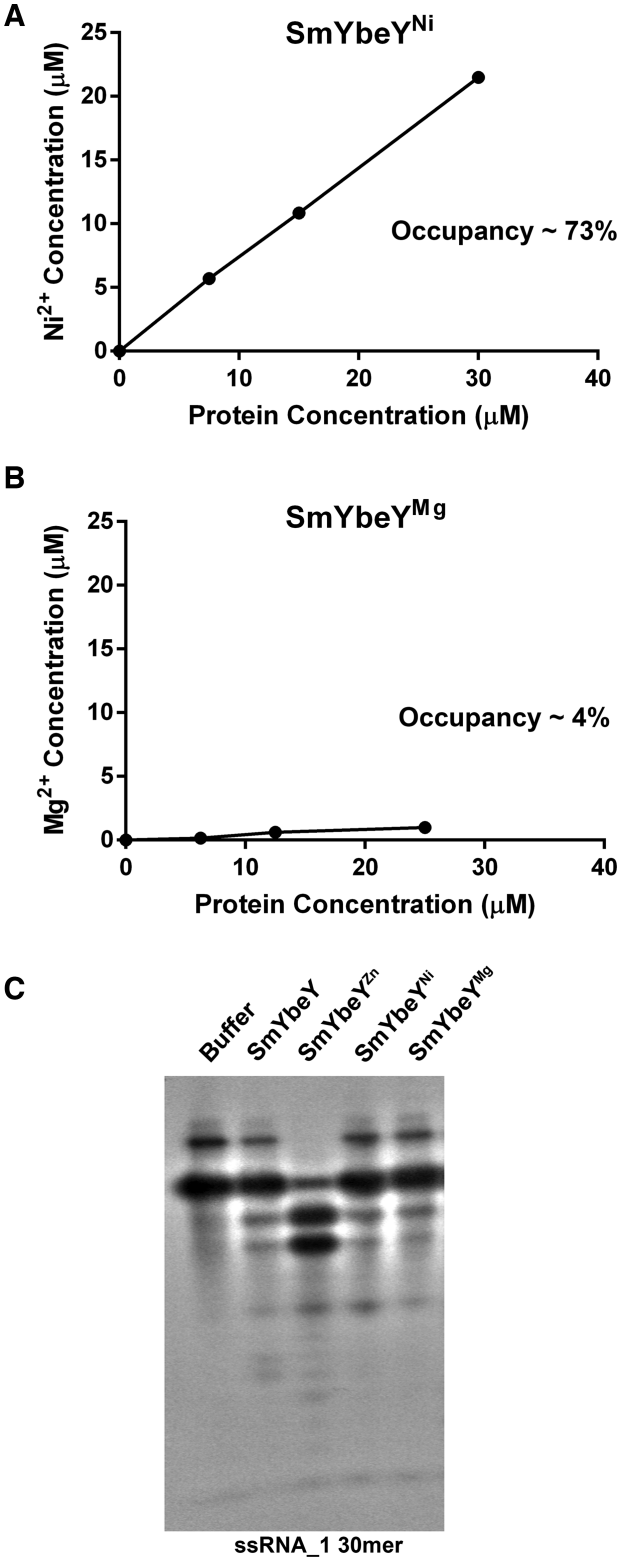
Nickel binds SmYbeY and inhibits its activity. (**A**) Nickel concentration in SMYbeY^Ni^ was calculated by subjecting different concentrations of protein sample to ICP-MS analysis. (**B**) Magnesium concentration in SMYbeY^Mg^ was calculated by subjecting different concentrations of protein sample to ICP-MS analysis. (**C**) ^32^P labeled single-stranded RNA substrate (ssRNA_1) was incubated for 1 h at 37°C with the Buffer or 25 μM SmYbeY or 25 μM SmYbeY^Zn^ or 25 μM SmYbeY^Ni^ or 25 μM SmYbeY^Mg^. The reaction mixture was run on a 20% polyacrylamide TBE-Urea gel. The panel is representative of at least three independent experiments.

We then repeated the SmYbeY purification protocol, but with the addition of Ni^2+^ during overproduction (0.1 mM Ni^2+^) and in the lysis buffer (10 mM Ni^2+^). The protein was then purified through Strep-tag purification and size exclusion chromatography using buffers lacking excess metals as described in Materials and Methods. The protein was then dialyzed into metal-free water using a buffer exchange column. SmYbeY purified this way (SmYbeY^Ni^) was subjected to ICP-MS and the concentration of protein-bound nickel was determined. Ni^2+^ occupancy in SmYbeY^Ni^ was thus calculated to be ∼73% (Figure 4A). This result indicates the nickel is another metal that is capable of binding to SmYbeY protein. This is in agreement with the crystal structure of YbeY showing a coordinating Ni^2+^ ion since the purification of that protein involved the use of nickel affinity columns (22). In a similar fashion, SmYbeY was produced and lysed in the presence of excess Mg^2+^. SmYbeY purified this way (SmYbeY^Mg^) had the Mg^2+^ occupancy of ∼4% (Figure 4B). We then tested if the presence of Ni^2+^ or Mg^2+^ in the protein could support RNase activity. RNA degradation assays demonstrate that RNase activity of SmYbeY^Ni^ or SmYbeY^Mg^ was less than that of SmYbeY^Zn^ and indistinguishable from the activity of YbeY purified with no metal supplementation (Figure 4C). Taken together, our results indicate that native YbeY is a Zn^2+^-containing protein that does not normally bind Mg^2+^.

### Loss of YbeY results in an rRNA processing defect in *Sinorhizobium meliloti*

**Figure 5.**
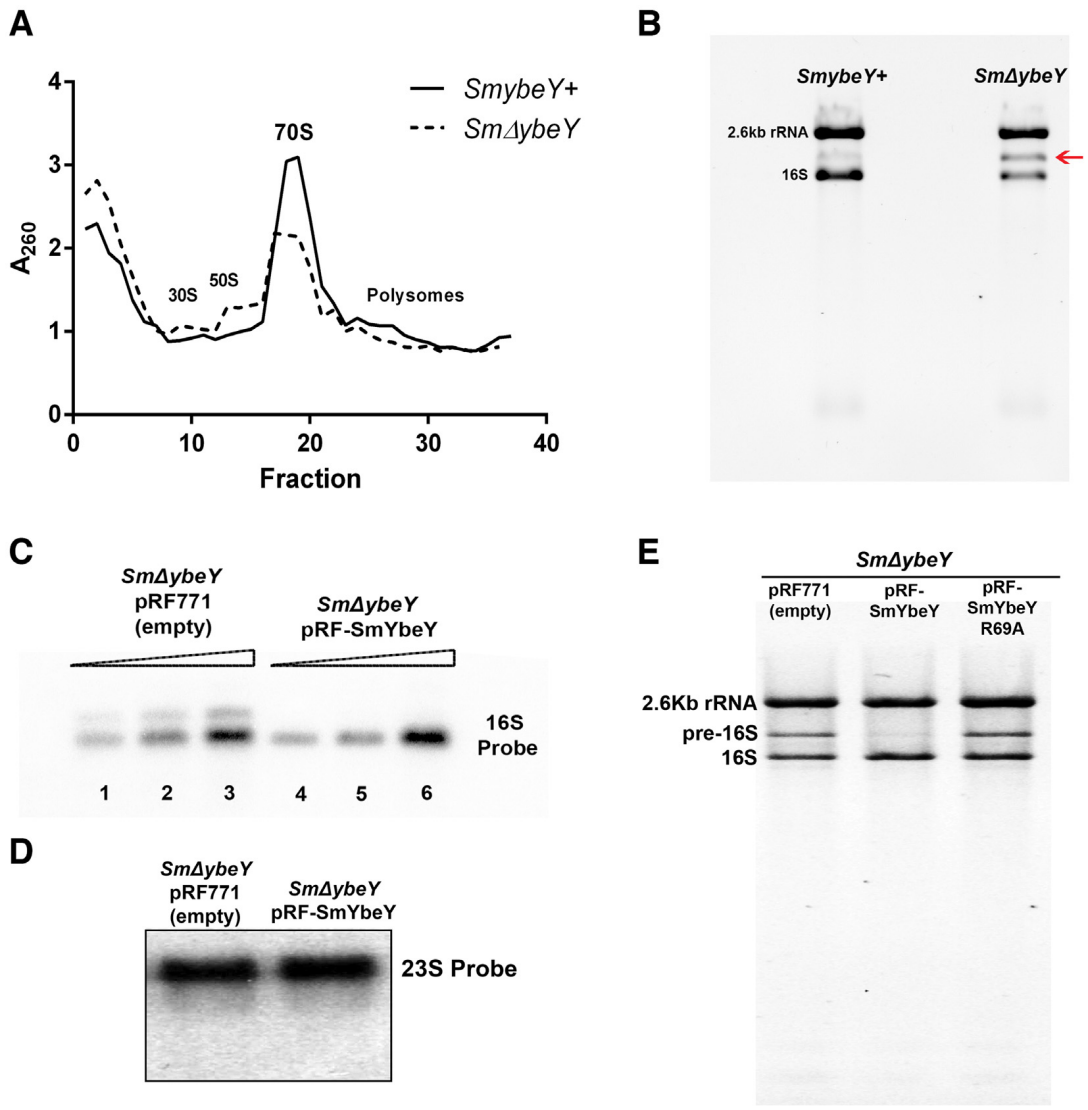
Ribosomal defects due to loss of YbeY in *S. meliloti*. (**A**) Cell lysates from the exponentially growing cells were analyzed in a sucrose gradient as described in Material and methods. The *S. meliloti* strain lacking YbeY (dashed line) shows a reduced amount of 70S ribosomes and an increase in the 30S and 50S ribosomal particles compared to the wild type *S. meliloti* (Solid line). (**B**) Total RNA prepared from the wild type *S. meliloti* (*SmybeY+*) and *S. meliloti* lacking YbeY (*SmΔybeY*) were separated by an agarose/ Synergel electrophoresis. *SmΔybeY* strain accumulates increased levels of an unknown rRNA band (Red arrow) compared to the *SmybeY+* strain, intermediate in size between the 2.6Kb RNA and the 16S RNA. (**C**) Northern Blot using 16S specific ^32^P labeled probe detects the unknown band in the *SmΔybeY* strain carrying the empty vector (pRF771) but not in the *SmΔybeY* strain carrying the SmYbeY expressing plasmid (pRF-SmYbeY). Increasing concentrations of RNA (1, 2.5 and 5 μg) used from lane 1 to lane 3 and from lane 4 to lane 6. (**D**) Northern blot using the 23S specific probe fails to detect the unknown rRNA band supporting the conclusion that the unknown band in *SmΔybeY* strain is a 16S rRNA species. (**E**) Total RNA, prepared from the *S. meliloti* lacking YbeY (*SmΔybeY*) carrying plasmids that are empty (pRF771) or expressing wild type SmYbeY (pRF-SmYbeY) or SmYbeY R69A (pRF-SmYbeY R69A), were separated by an agarose/Synergel electrophoresis. *SmΔybeY* strain complemented with wild type SmYbeY did not accumulate pre-16S rRNA while complementation with SmYbeY R69A accumulated pre-16S rRNA.

The highly pleiotropic phenotypes of *S. meliloti* and *E. coli ΔybeY* mutants resemble each other, suggesting that many physiological functions of YbeY are conserved between the two organisms (4). Similar to *E. coli*, loss of YbeY results in a slow-growth phenotype in free-living *S. meliloti*. When grown in LB media, the growth of an *S. meliloti* Δ*ybeY* mutant (*SmΔYbeY*), as measured by OD_600 nm_, lags significantly over a period of 24 hours compared to the wild type (*SmYbeY+*) (Supplementary Figure S7A). In both *E. coli* and *S. meliloti*, loss of YbeY results in sensitivity to DNA damaging agents such as UV, to oxidizing agents such as hydrogen peroxide, to agents causing membrane stress such as deoxycholate, and to various antibiotics (2,4,16). In *E. coli*, loss of YbeY also leads to a defect in ribosome assembly causing accumulation of the 30S and 50S precursors (4,45). In order to determine the role of YbeY in ribosome assembly in *S. meliloti*, we measured the relative amounts of ribosome particles present in wild type *ybeY^+^* and *ΔybeY S. meliloti* strains. Polysome profile obtained from the *A*_260 nm_ absorbance of the sucrose gradient fractions of the cell lysate indicate that *S. meliloti ΔybeY* mutant has a markedly reduced fraction of assembled 70S ribosomes and increased free 30S and 50S ribosomal subunits compared to the *ybeY^+^* parental strain (Figure 5A). This result suggests that, like the *E. coli ΔybeY* strain (4), the *S. meliloti ΔybeY* strain has numerous defective ribosomal subunits that fail to assemble into 70S ribosomes.

One of the most striking phenotypes due to loss of YbeY in *E. coli* is the accumulation of a 17S precursor of 16S ribosomal RNA (rRNA) (4). The 17S rRNA includes 115 extra nucleotides at the 5′ end and 33 extra nucleotides at the 3′ end of the 16S rRNA. In contrast, Saramago *et al.* (31) reported that no 16S precursors accumulate in an *S. meliloti* Sm2B3001 *ΔybeY* strain (31). This result was unexpected given the number of common phenotypes observed between *E. coli ΔybeY* and *S. meliloti ΔybeY* strains. Therefore, we isolated total RNA from our laboratory *S. meliloti* strain 1021 (previously known as Rm1021) and its *ΔybeY* derivative and studied the rRNA profile by agarose/Synergel electrophoresis. This experiment revealed the presence of an unknown rRNA band migrating between the 2.6 kb-like 23S rRNA species and the 16S rRNA (Figure 5B). Since this finding was clearly different from what was reported by Saramago *et al.* (31), we repeated the experiment with the SM2B3001 strains used by those authors and were able to see the unknown band in the SM2B3001 *ΔybeY* derivative as well (Supplementary Figure S8). This result confirms the existence of an unknown rRNA product in an *S. meliloti ΔybeY* independent of the particular strain used. We hypothesized that the differences between the two studies might be due to the media and experimental conditions used. We consistently used Luria Bertani (LB) media for our experiments (See Material and methods) while Saramago *et al.* (31) used a minimal media composition (referred to as MM in this study). To test whether if the differences are due to growth media, we extracted the total RNA from the SM2B3001 strains grown in MM. The total rRNA profile, still included the unknown band, but in somewhat reduced amount, (Supplementary Figure S8), thereby offering a possible explanation for why this phenotype was not observed in the previous study.

### Unknown band in the *SmΔybeY* strain is a 16S rRNA precursor

The accumulation of the unknown band longer than the 16S rRNA in the *S. meliloti ΔybeY* strain suggests that there is likely a 16S rRNA processing defect similar to *E. coli ΔybeY*. However, analysis of total RNA lengths in an RNA fragment analyzer (Agilent) revealed that the unknown band was much longer (∼ >1800nts) than the 17S rRNA precursor seen in *E. coli*. In order to find the identity of the unknown band, we performed a Northern blot analysis using probes specific to mature 16S or 23S rRNA. When using the 16S specific probe, we observed two bands in the RNA extracted from *SmΔybeY* pRF771 *(*empty vector) strain (Figure 5C). On the other hand, the upper band was not detectable when YbeY is expressed from a plasmid in the *SmΔybeY* pRF-YbeY strain (Figure 5C). When a 23S specific probe was used, there was only one band in both the *SmΔybeY* pRF771 and *SmΔybeY* pRF-YbeY strains (Figure 5D). This result shows that the unknown band that accumulates in *S. meliloti ΔybeY* strain is, in fact, a 16S rRNA precursor species and will be hereafter referred to as pre-16S rRNA. The ability of the phenotype to be complemented *in trans* shows that the pre-16S species accumulates due to the absence of YbeY. Since the mutation of R69 residue resulted in the reduction in SmYbeY enzymatic activity, we then tested if this mutation impairs function *in vivo*. We complemented the *SmΔybeY* strain with plasmid expressing SmYbeY R69A (pRF-SmYbeY R69A). SmYbeY R69A failed to prevent the accumulation of the pre-16S rRNA (Figure 5E). This result suggests that R69A is a loss of function mutation of YbeY *in vivo*.

### Pre-16S rRNA is longer in alpha-proteobacteria than in gamma-proteobacteria

**Figure 6.**
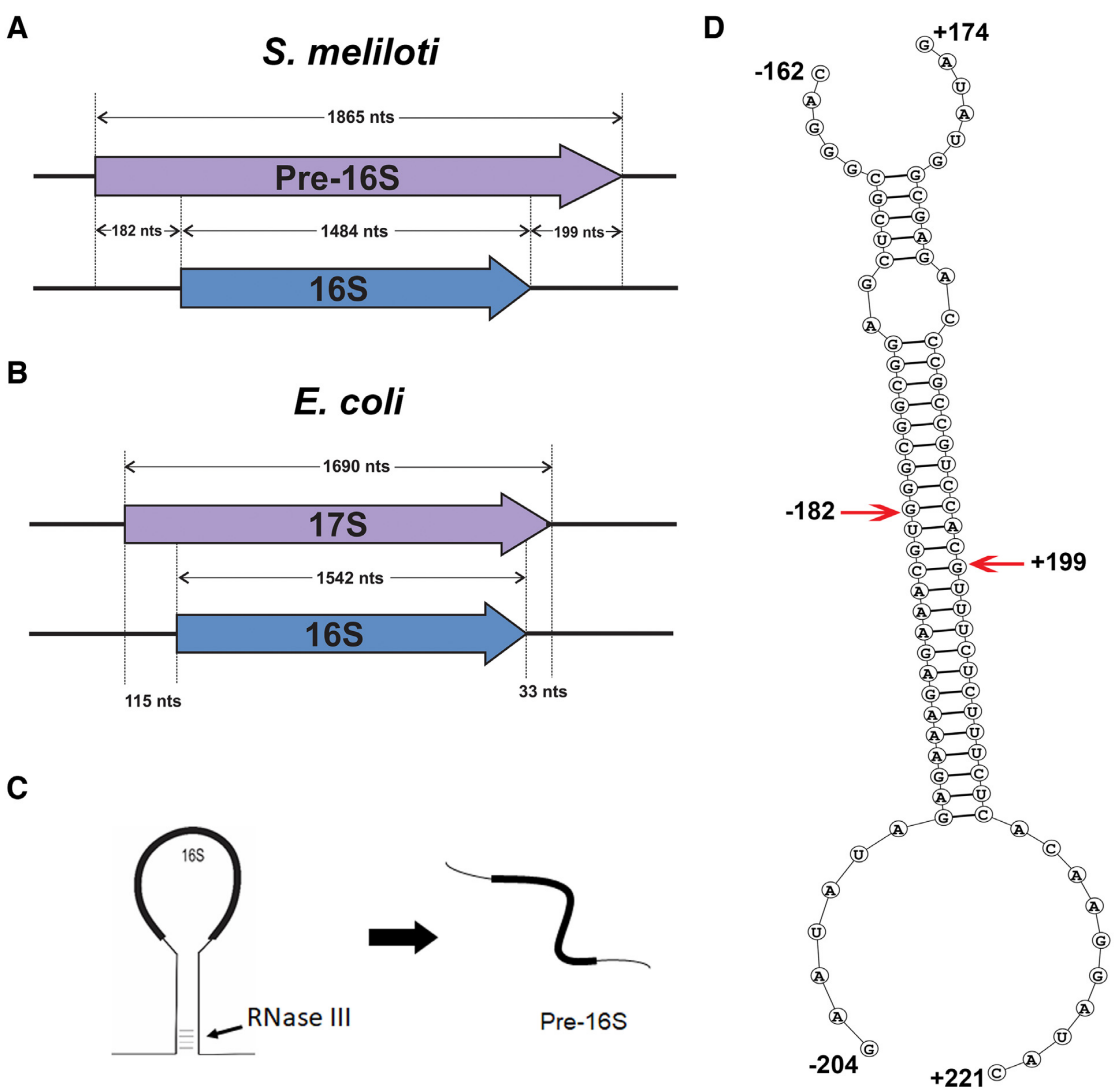
Characterization of the pre-16S band. (**A**) The pre-16S RNA was isolated by gel excision and the RNA ends were determined using the RACE protocol described in Materials and methods and FIG S8. Locations of the pre-16S (purple) ends are represented in comparison with the mature 16S rRNA (blue). (**B**) Length of the 17S rRNA (purple) relative to 16S (blue) in *E. coli* is shown. Size of the 17S in *E. coli* is relatively smaller compared to the pre-16S of *S. meliloti*. (**C**) A simplified view of RNase III cleavage of the rRNA primary transcript. RNase III cleaves the double-stranded stem structure formed by the nascent primary rRNA transcript thereby releasing the pre-16S RNA. (**D**) Secondary structure of the rRNA region with the pre-16S ends indicated with red arrows. Analysis of the ends reveals that they originated from the RNA regions that are complementary to each other and are capable of forming a 31 bp stem with two mismatches in between.

In order to characterize the pre-16S rRNA, we determined the 5′ and 3′ ends of this species. Since we had established that the band of interest is a 16S species, we were able to use a shortened RACE protocol using gel-purified pre-16S as a template and 16S sequence-specific primers to determine the rRNA ends (Supplementary Figure S9). By this method, we determined that the 5′ end of the pre-16S rRNA is located at 182 nucleotides upstream of the mature 5′ end of 16S and the 3′ end of the pre-16S to be at 199 nucleotides downstream of the mature 3′ end of 16S (Figure 6A). The pre-16S in *S. meliloti* has about 1865 nucleotides in total, 175 nucleotides longer than *E. coli* 17S rRNA (Figure 6B). In *E. coli*, the 17S precursor is formed after the RNase III protein cleaves the double-stranded stem region of the 16S stem-loop structure of the rRNA primary transcript (46) (Figure 6C). Therefore, the sequences at which the RNase III cuts the 16S rRNA primary transcript are complementary to each other (47). This led us to examine the pre-16S cut sites for complementarity in *S. meliloti*. Similar to *E. coli*, the sequence surrounding the cut sites at the 5′ and 3′ ends are complementary to each other and are capable of forming a stem structure with 24 base pairs (Figure 6D). Furthermore, the cut sites are three nucleotides apart on each strand. RNase III protein is known to make two cuts on the stem structure with primarily two or sometimes three nucleotides structurally apart on each strand (48). This result suggests that the pre-16S product in *S. meliloti* and the 17S rRNA in *E. coli* are products of a similar RNase III enzymatic cleavage event.

### Pre-16S rRNA is longer in alpha-proteobacteria compared to gamma-proteobacteria

**Figure 7.**
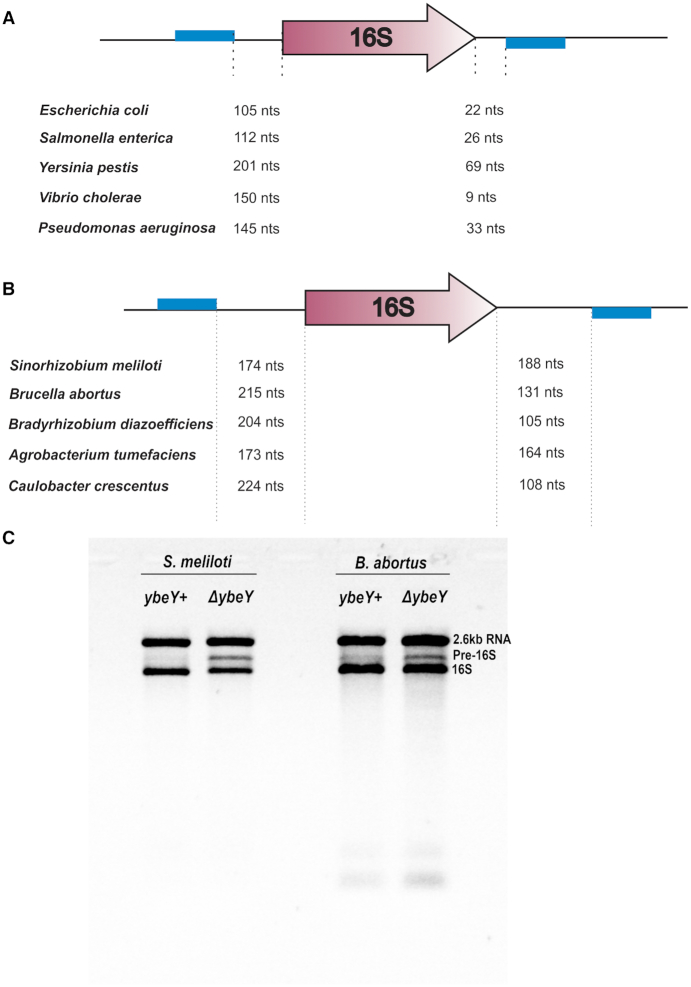
Comparison of the pre-16S rRNA between alpha- and gamma-proteobacterial candidates. (**A**) Location of the sequences having the longest complementarity (Blue) at either side of the 16S (purple) in gamma-proteobacteria. (**B**) Similar to A., but in alpha-proteobacteria candidates indicating the increase in distance of these sequences from the 16S gene. (**C**) Loss of YbeY in *B. abortus* results in the accumulation of pre-16S species. Total RNA prepared from the wild type *B. abortus* (*BaybeY+*) and *B. abortus* lacking YbeY (*BaΔybeY*) were separated by an agarose/ Synergel electrophoresis. *BaΔybeY* strain accumulates increased levels of pre-16S rRNA band compared to the *BaybeY+* strain, intermediate in size between the 2.6 kb RNA and the 16S RNA.

We then investigated why the pre-16S from *S. meliloti* is longer than the *E. coli* 17S. Since the canonical RNase III cleavage takes place at the complementary sequence on either side of the 16S rRNA, we simply looked for those regions that are capable of forming a stem structure longer than 15 bp in other candidates from alpha-proteobacteria and gamma-proteobacteria. The distance between the last nucleotide of the complementary region and the 5′ end of 16S rRNA and the distance between the 3′ end of the 16S rRNA and the first nucleotide of the complementary region were calculated. Our analysis shows that the complementary regions are further apart in alpha-proteobacteria (Figure 7B) compared to gamma-proteobacteria (Figure 7A). Specifically, the distance between the 3′ end of rRNA and the complementary region downstream is significantly shorter in gamma-proteobacteria compared to alpha-proteobacteria. This result indicates that there is sequence divergence between the two groups at either side of 16S rRNA processing resulting in the change in the location of the stem structure sequences. Despite this difference, *ΔybeY* strains that belong to both groups accumulate the product of RNase III cleavage step in 16S rRNA processing suggesting that the YbeY protein is functionally conserved.

In order to verify experimentally whether the pre-16S rRNA is longer in another alpha-proteobacteria, we analyzed the human pathogen, *Brucella abortus*, which also belongs to this group. It has been previously shown that YbeY is required for the ability of *B. abortus* to survive and replicate inside macrophages (27). The study also showed that *B. abortus* was defective in its ability to colonize mouse spleen signifying the importance of YbeY in *B. abortus* infection. However, the role of YbeY in rRNA processing in *B. abortus* has remained unknown. We analyzed the total RNA from *B. abortus* wild-type and *ΔybeY* strains and the result clearly shows that *B. abortus ΔybeY* strain also accumulates pre-16S rRNA precursor similar to *S. meliloti ΔybeY* strain (Figure 7C). The length of the precursor was similarly longer in *B. abortus* as predicted by our analysis. This result suggests that the stem region of the 16S precursor is further away from the mature 16S ends in *B. abortus* as well. This result supports our model that even though the length of the precursor is different between alpha-proteobacteria and gamma-proteobacteria, the cells lacking YbeY in both groups accumulate a similar enzymatic cleavage product that has not undergone further processing.

### Phenotypes of *E. coli ΔybeY* strain can be complemented by *S. meliloti* YbeY protein and vice versa

**Figure 8.**
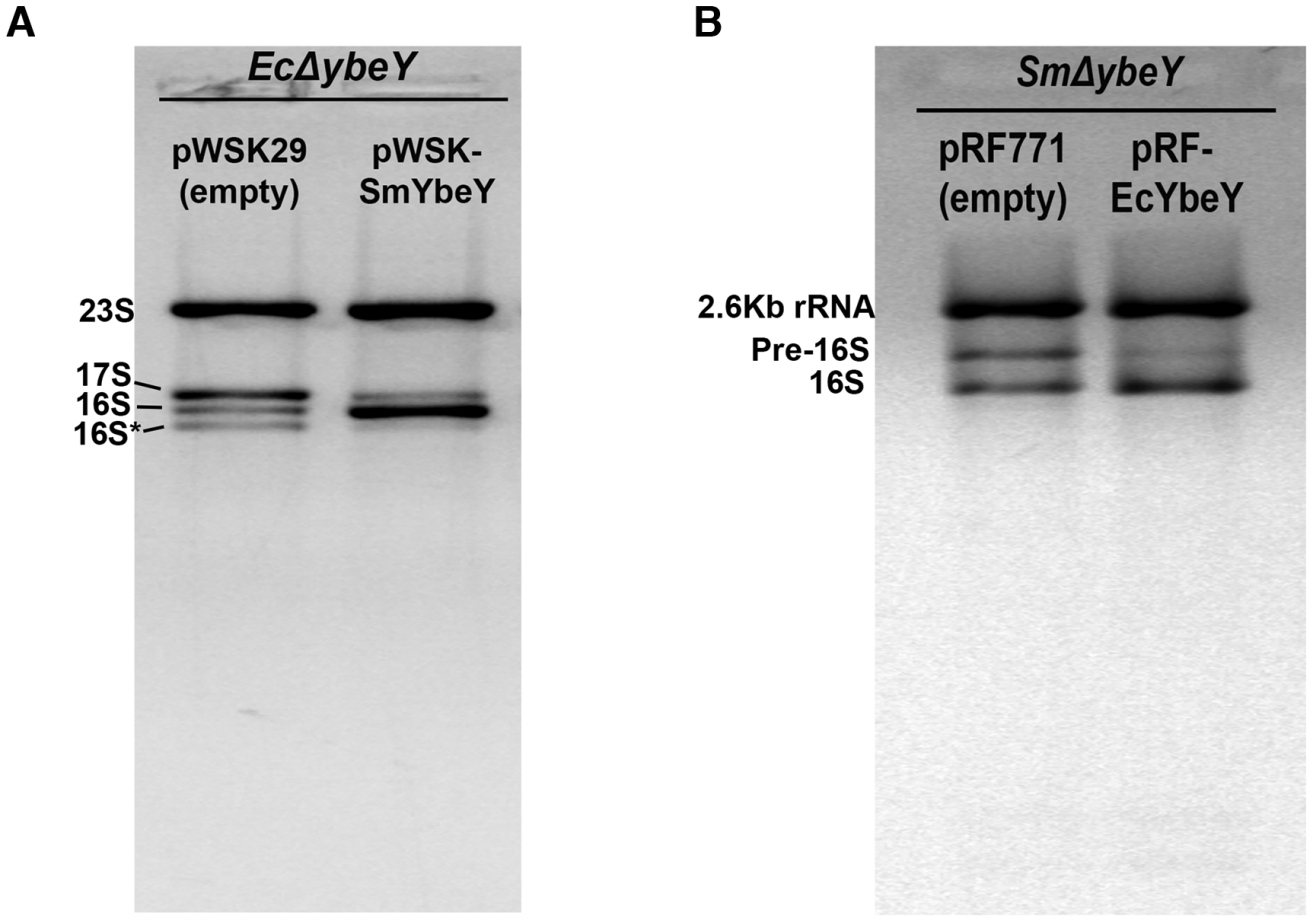
(**A**) SmYbeY complements the rRNA processing defect in *EcΔybeY*. Total RNA was extracted from the *E. coli* strain lacking YbeY (*EcΔybeY*) and carrying either the empty vector (pWSK29) or the plasmid expressing *S. meliloti* YbeY (pWSK-SmYbeY) and were subjected to electrophoresis in an Agarose/Synergel mix gel. The accumulation of the precursor 17S and the 16S* degradation product were reduced in the *EcΔybeY* strain expressing SmYbeY. (**B**) EcYbeY complements the rRNA processing defect in SmYbeY. Total RNA extracted from the *S. meliloti* strain lacking YbeY (*SmΔybeY*) and carrying either the empty vector (pWSK29) or the plasmid expressing *E. coli* YbeY (pWSK-EcYbeY) were subjected to electrophoresis similar to the A panel. The accumulation of the pre-16S rRNA product was reduced in the *SmΔybeY* strain expressing EcYbeY.

In order to further establish that the functions of YbeY are conserved between *E. coli* and *S. meliloti*, we performed *in vivo* heterologous complementation experiments. Firstly, we expressed the *S. meliloti* YbeY protein (SmYbeY) in the *E. coli* strain lacking YbeY protein (*EcΔybeY*). Growth analysis at 30°C indicates that the *EcΔybeY* pRF-SmYbeY strain has significantly improved growth rate compared with the *EcΔybeY* strain with only the empty vector (pWSK29) (Supplementary Figure S7B). Total RNA profile shows that the *EcΔybeY pWSK29* strain accumulates 17S rRNA precursor and the 16S* band (Figure 8A). The 16S* is a 16S degradation band that accumulates in *E. coli* and few other bacteria (4,13,14,16,17,23,26). On the other hand, the *EcΔybeY* pWSK-SmYbeY strain has markedly reduced 17S rRNA precursor and a significantly increased amount of mature 16S rRNA. This result shows that SmYbeY is capable of functionally complementing the loss of YbeY in *E. coli* with respect to 16S rRNA processing. Conversely, we also expressed the *E. coli* YbeY protein (EcYbeY) from a plasmid (pRF-EcYbeY) in the *SmΔybeY* strain. Growth analysis at 30°C shows that the *SmΔybeY* pRF-EcYbeY strain has significantly improved the growth rate compared to the *SmΔybeY* strain with the empty vector (pRF771) (Supplementary Figure S7C). Total RNA profile indicates the *SmΔybeY* pRF-EcYbeY strain has reduced pre-16S rRNA accumulation compared to *SmΔybeY* pRF771 strain (Figure 8B). This result indicates that EcYbeY is capable of complementing the loss of YbeY in *S. meliloti* with respect to 16S rRNA processing. Taken together, these results establish that YbeY protein is functionally highly conserved between *S. meliloti* and *E. coli*. In addition, this result also suggests that YbeY protein from either of the hosts are capable of interacting with their partner proteins from the other host to perform its functions *in vivo* since evidence suggests that the relatively non-specific YbeY is directed to the 3′ end of the 16S rRNA precursor via contacts with proteins such as S11 and Era (16).

### Heat stress has a modest effect on rRNA processing in *S. meliloti* lacking YbeY

**Figure 9.**
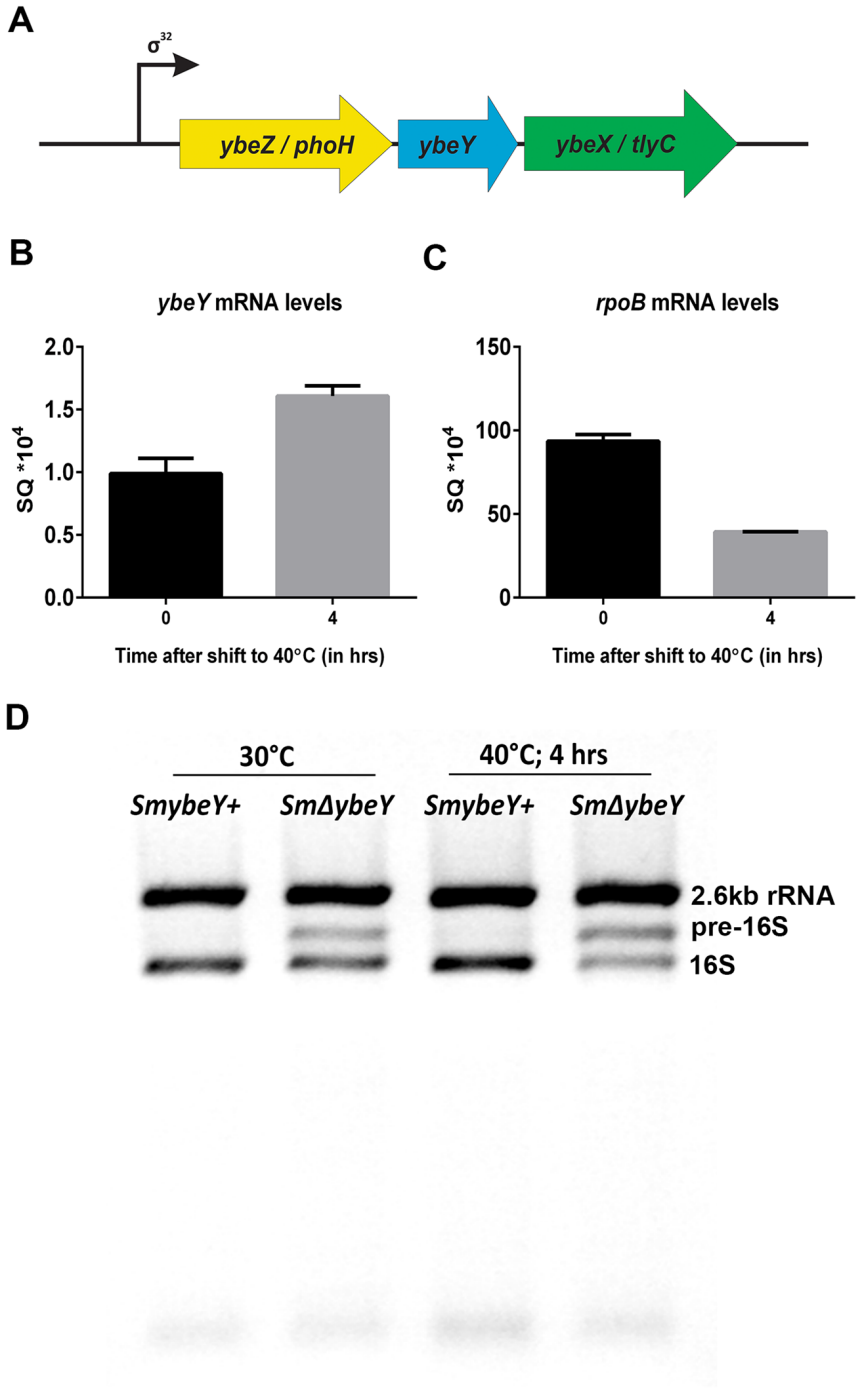
(**A**) Organization of the YbeY gene locus. In both *S. meliloti* and *E. coli*, *ybeY* gene (in blue) is located between the *ybeZ* gene (in yellow; also known as *phoH*) and the *ybeX* gene (in green; also known as *tlyC*) in the YbeZYX operon. (**B** and **C**) Wild type *S. meliloti* cells were grown at 30°C till mid-exponential phase and then subjected to 40°C for 4 h. Samples were collected before and after the shift to high temperature. Total RNA was extracted and the mRNA levels of *ybeY* (B) and *rpoB* (C) were determined using qPCR. (**D**) Both Wild type and *ybeY* mutant *S. meliloti* cells were subjected to heat stress as described in B and C. Total RNA was extracted and subjected to electrophoresis in an agarose/synergel mix gel.

In *E. coli, ybeY* is part of the *ybeZYX* operon whose expression is induced by the *rpoH* encoded sigma factor σ^32^ as part of the heat shock response. A previously reported microarray analysis of *S. meliloti* subjected to heat shock at 40°C for 30 min revealed several genes that are upregulated (49). However, *ybeY* was not identified to be one of the induced genes in that study. In *S. meliloti*, there are two RpoH like proteins, RpoH1, and RpoH2 (50,51). RpoH1 has been shown to be required for symbiosis (52). RpoH2 is thought to be expressed later during heat shock while RpoH1 is expressed early (53). In another study, *S. meliloti* wild-type and mutants of the *rpoH* genes were shifted to 42°C and the gene expression changes were analyzed using a microarray (54). This study also did not report finding *ybeY* as one of the heat-induced genes. However, the study did identify *ybeX/tlyC* (*Smc00112*) as being induced slightly less than two-fold. *S. meliloti ybeY* gene is located in the same operon as *ybeZ* and *ybeX* homologs designated as *phoH* and *tlyC* respectively (Figure 9A). Hence, a modest induction of YbeY after heat shock could still occur. Therefore, we performed the heat shock to a longer time of 4 hours and tested if the *ybeY* expression is affected. Our result shows the expression of *ybeY* increases after heat shock (Figure 9B) to ∼1.7-fold compared to the time before heat shock. As a control to validate our mRNA sample, we measured the *rpoB* mRNA levels and found that the *rpoB* levels are reduced after heat shock (Figure 9C). In *E. coli*, YbeY is induced to over four-fold after heat shock of 42°C for 20 min (55). This result suggests that the although the expression of *ybeY* might be regulated similarly in *S. meliloti* like *E. coli*, the induction could be milder in *S. meliloti*.

## DISCUSSION

The SmYbeY used in this study was expressed in an *rna pnp* derivative of the *E. coli* strain BL21(DE3) strain that lacks both RNase I and polynucleotide phosphorylase. The strain also contains the pRARE2 plasmid, which carries seven rare codon tRNA genes and thus should help with the expression of *S. meliloti ybeY*, which contains eleven rare codons not present in *E. coli ybeY*. Our SmYbeY was purified by a combination of Strep-tactin affinity purification and size exclusion chromatography. In contrast, the preparation of SmYbeY used by Saramago *et al.* (31), was obtained by expressing a histidine-tagged derivative in *E. coli* strain BL21(DE3), followed by a purification procedure involving the use of a Ni^2+^-charged column and anion exchange chromatography. A concern about the use of a Ni^2+^-chelating column to purify any YbeY ortholog is that the native metal could be displaced by Ni^2+^. In one study that utilized a nickel column for purification of YbeY, the crystal structure revealed the presence of Ni^2+^ ion in the active site of the enzyme although the occupancy was not 100% (22). In our lab's past analyses of YbeY biochemistry, the *E. coli, V. cholerae*, and human YbeY orthologs were purified as fusions to maltose-binding protein (MBP) followed by TEV cleavage so as to avoid exposure to the metals like nickel/cobalt used in metal affinity purification (13,15,30). We also avoided the use of EDTA which can result in the isolation of a protein that lacks any metal, as in the previously reported cases of *Aquifex aeolicus* and *Haemophilus influenzae* YbeYs (20,56). All our previous preparations of YbeY orthologs, purified as fusions to MBP, displayed RNase activity without any added metal, as did the SmYbeY preparations used in this study (13,15,30). Since Saramago *et al.*, reported no RNase assays carried out in the absence of these divalent metal cations (31), it was not possible to assess whether the native metal ion of SmYbeY in their preparations might have been displaced by Ni^2+^ during metal affinity chromatography. The high concentrations of metals used in the Saramago *et al.* enzyme assays (31) is also problematic because unbound metals have been shown to induce auto cleavage of RNA (57–59).

Saramago *et al.*’s conclusion that *S. meliloti* YbeY degrades both single and double-stranded RNA substrates (31) was surprising, yet interesting in the context of the intermediate that accumulates in the absence of YbeY. The pre-16S intermediate formed after RNase III cleavage has residual complementary residues that can remain double stranded. In principle, the processing the 3′-terminus of the 16S rRNA could involve the action of a double strand endoribonuclease, however the participation of other proteins can make the processing possible by a single strand endoribonuclease. For example, Tu *et al.* (60) have shown that the 3′-terminus of the 16S precursor is bound by the Era protein in a manner that leaves three nucleotides of the mature 3′-terminus protruding out of the Era/16S rRNA complex. It has been suggested that Era binding could induce conformational changes that may facilitate the activity of RNases that remove the extra nucleotides (61).

Our results suggest a simple alternate explanation for the origin of the SmYbeY double-strand endoribonuclease activity reported by Saramago *et al.* (31) is likely to be contaminating *E. coli* RNase III activity present in the preparations of *S. meliloti* YbeY. This possibility is supported by the observation that the reported cut sites of YbeY preparation of Saramago *et al.*, on R1.1 substrate being the same as the known RNase III cut sites (31,43). The metal requirement reported for the double-strand activity of YbeY is reported to be magnesium and was inhibited by calcium which are the known characteristics of RNase III (62). The correlation between the zinc occupancy and activity in the protein sample together with the structural predictions strongly suggests that YbeY is a zinc-dependent enzyme. We were unable to detect a double-strand endonuclease activity from YbeY alone under normal experimental conditions where single-strand activity can be easily detected. YbeY has a low specific activity so contamination by Mg^2+^/Mn^2+^-dependent RNases of higher specific activity is a significant concern. Because of the differences in the *E. coli* strains used to express SmYbeY and the very different strategies employed for protein purification, it is likely that any minor amounts of contaminating RNases could be different between the SmYbeY preparation of Saramago *et al.* (31) and the SmYbeY described here.

Both the *S. meliloti* pre-16S product identified in this study and the 17S rRNA in *E. coli* described previously contain the residual double-stranded region after the RNase III cleavage step. Enzymes implicated to act on this substrate are RNase E and RNase G at the 5′ terminus of 16S, the four exoribonucleases and YbeY at the 3′ terminus of 16S. All of these enzymes were shown to act only on single-stranded substrate except for the processive exoribonuclease RNase R which can act on RNA secondary structures provided there is a 3′ overhang of at least seven nucleotides in length (63,64). The absence of double-strand activity *in vitro* for YbeY does not exclude the possibility that YbeY might act with one or more partner proteins on such substrates. One partner could be RNase R which has been shown to be able to function with YbeY in 70S ribosome quality control (13). Another candidate could be YbeZ, the upstream gene product of the *ybeZYX* operon which possesses the helicase like NTPase domain. YbeZ has been shown to interact with YbeY however its function is currently unknown (16). Presence of minor quantities of YbeY binding partners in the protein preparations could enable the complex to act on double-stranded substrates. Absence of YbeY does not completely eliminate the formation of the mature 16S rRNA which suggests that the multiple processing pathways exist for the maturation process. Utilization of the YbeY dependent or independent pathway choice could be influenced by the secondary structures of the pre-16S RNA intermediates.

In this study, we have also revisited the role of the highly conserved protein YbeY in *S. meliloti* following the report by Saramago et al. (31), that they could not detect any effect on the rRNA processing in *S. meliloti* lacking YbeY. Here, we show that absence of YbeY does, in fact, result in the accumulation of a 16S precursor. We attribute this difference in observations between the two studies to the strains and experimental conditions used. In fact, in our hands, the strain and experimental condition used by Saramago *et al.* (31), gave us the least accumulation of the pre-16S species. Similar to the 17S species in *E. coli*, the pre-16S species in *S. meliloti* is more likely to be an RNase III cleavage product given the location of the cut sites. It is interesting to note that the location of this cut site on the primary rRNA transcript has diverged between the alpha-proteobacteria compared to the gamma-proteobacteria. However, the commonality of the cleavage products observed in the strains lacking YbeY in both groups suggests strong conservation for the role of YbeY in rRNA processing. This idea is further supported by the ability of YbeY proteins from *E. coli* and *S. meliloti* to reciprocally complement the rRNA processing defects in either organism lacking YbeY. These observations suggest that the structure of the pre-16S substrate bound by YbeY and its partners must be similar in alpha- and gamma-proteobacteria enabling their processing by YbeY from either groups. The functional significance of the divergence in the pre-16S length remains to be investigated. On the other hand, the effect of heat shock seems to have a minor effect on the rRNA processing defect of the *S. meliloti ΔybeY* strain. The induction of YbeY after heat shock also seems to be modest yet significant in comparison to the strong response to heat shock in *E. coli*. These results suggest that *S. meliloti* and possibly other alpha-proteobacteria are more resilient to heat stress in the absence of YbeY. These results are further supported by similar observations in a recent study on *Agrobacterium tumefaciens* (26).

## Supplementary Material

gkz1095_Supplemental_File
