# Supplementary material for: Sinorhizobium meliloti YbeY is a zinc-dependent single-strand specific endoribonuclease that plays an important role in 16S ribosomal RNA processing
Source: Nucleic Acids Res. 2019 Nov 28;48(1):332–48. doi: 10.1093/nar/gkz1095 (PMC6943124; doi:10.1093/nar/gkz1095)
Supplement: gkz1095_Supplemental_File [file gkz1095_supplemental_file.pdf]

SUPPLEMENTARY FIGURES

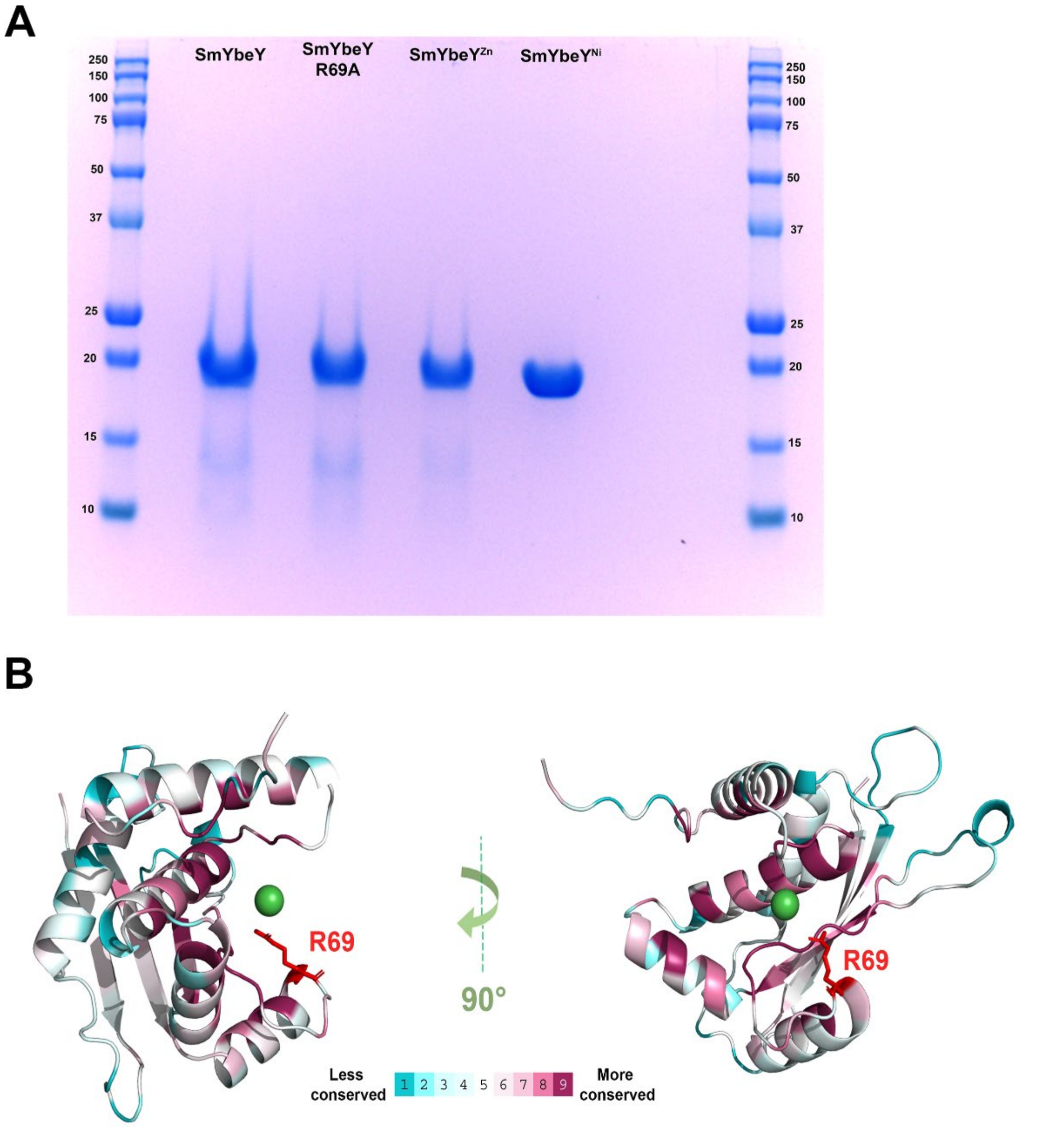

Figure S1. A. Protein homogeneity. Protein preparations (10µl of 0.7 mg/ml each) were run on 12% SDS-PAGE gel and stained with Coomassie blue showing that the contaminating proteins were undetectable. **B.** *S. meliloti* YbeY was homology modeled in SWISS-MODEL using *E. coli* YbeY structure (1XM5) as a template. The conservation scores (obtained from ConSurf) were projected for each residue as color from blue (less conserved) to purple (highly conserved) using PyMol. The location of the R69 residue mutated in this study is indicated as a red stick side chain. The location of the metal ion coordinated in the YbeY protein is indicated by the green sphere.

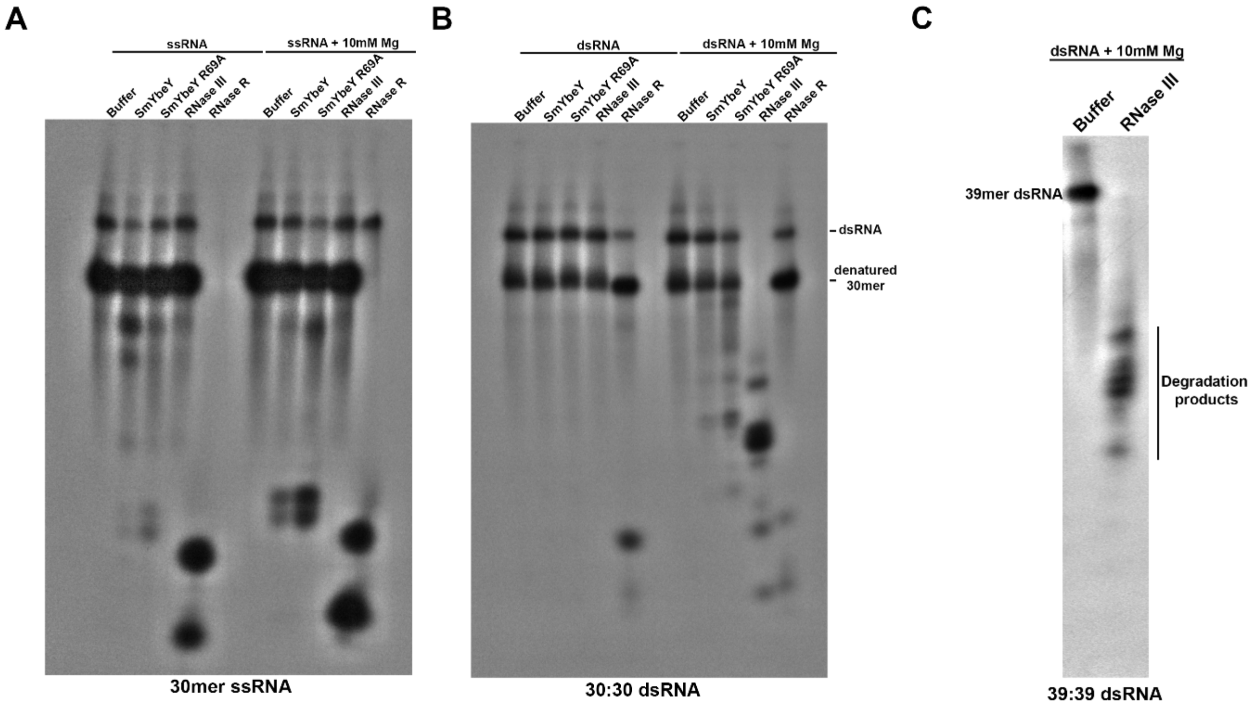

Figure S2. A. Degradation of ssRNA by YbeY and RNase R but not RNase III. **B.** Degradation of dsRNA (30:30) by RNase III but not by YbeY and RNase R. The indicated substrates are mixed with 25μM of indicated proteins in the presence or absence of 10mM Mg^{2+} and incubated at 37°C for 2 hrs. Panels are representative of at least three independent experiments. **C.** Degradation of dsRNA (39:39) by RNase III. The 39mer dsRNA was mixed with the Buffer or RNase III in the presence of 10mM Mg^{2+}.

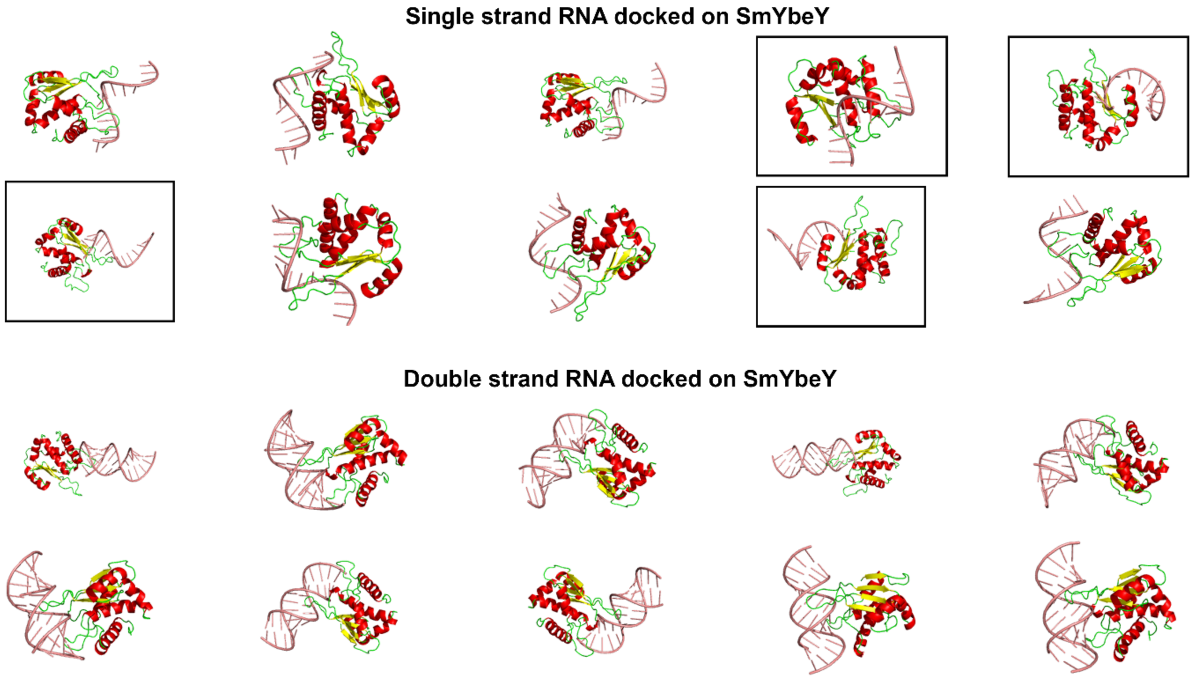

Figure S3. Single-strand RNA structure (extracted from PDB: 3ND3) was docked with the homology model of SmYbeY using ClusPro. The top 10 docking models are shown in the top panel. The models with the RNA strand fit into the active site of the YbeY protein were highlighted using a black square. Similarly, Double-stranded RNA structure (PDB: 5KVJ) was docked with the homology model of SmYbeY and the best models are indicated in the bottom panel. None of the models were able to fit a double-stranded RNA into the active site of SmYbeY.

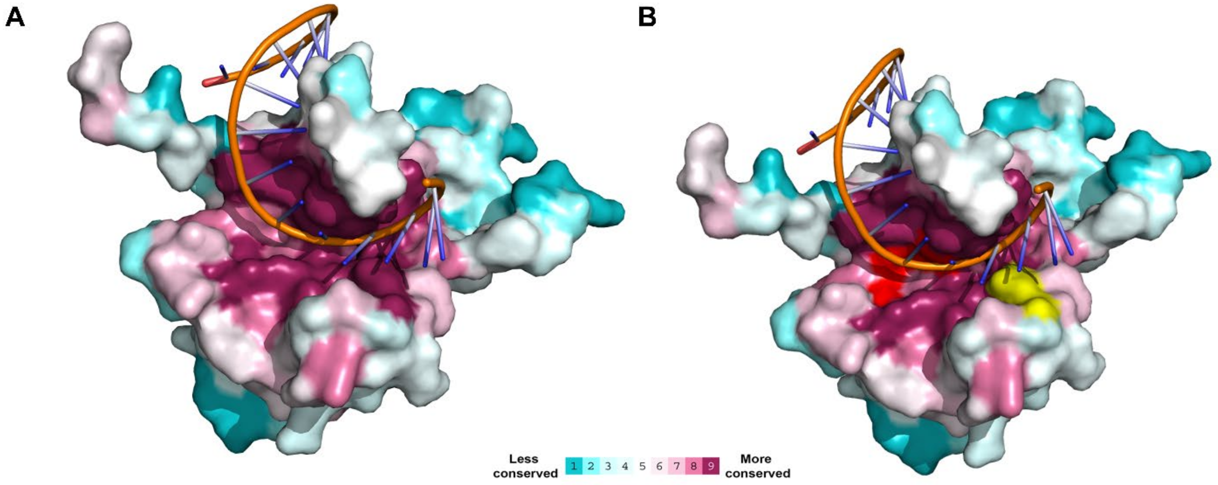

Figure S4. A. A model showing a good fit of the single-stranded RNA within the active site containing the highly conserved residues indicated in purple. Modeling was performed using ClusPro and visualized using PyMol. Conservation scores were obtained using the ConSurf tool. All models with double-strand RNA only fit near the less conserved residues (Blue) but not in the active site. **B.** Critical residues namely arginine R69 (yellow) and the histidine triad H119, H122 and H126 (Red) are highlighted.

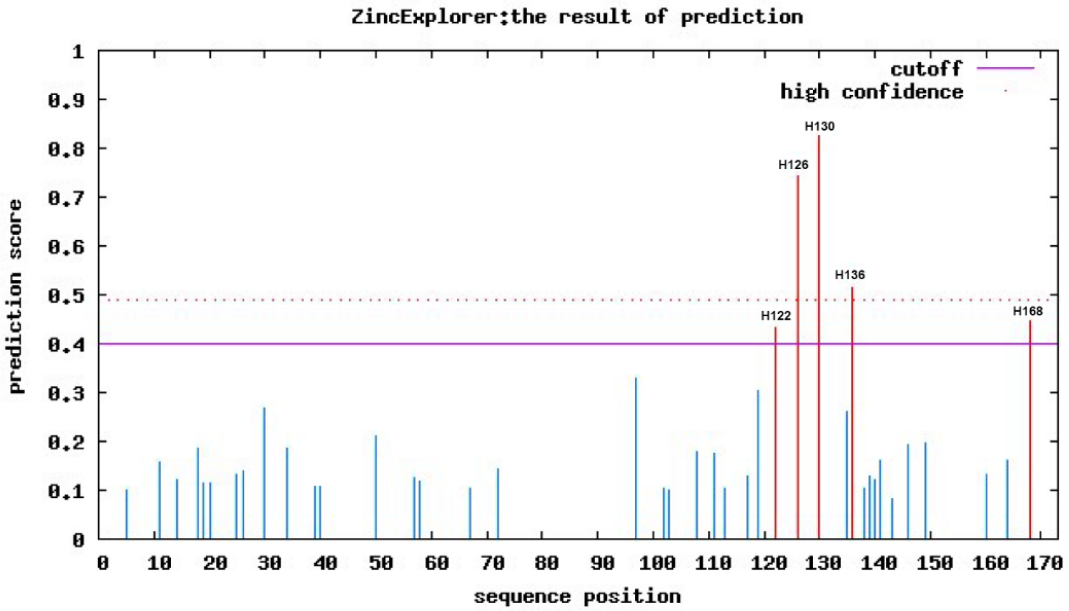

Figure S5. Zinc binding probabilities for each residue of SmYbeY were analyzed using ZincExplorer. Five residues (H122, H126, H130, H136 and H168) are predicted to possibly bind zinc as they had scores above the cutoff prediction score indicated by the purple line. Three of those residues (H126, H130 and H136) are highly likely to bind zinc as they have scores above the high confidence score indicated by the dotted line.

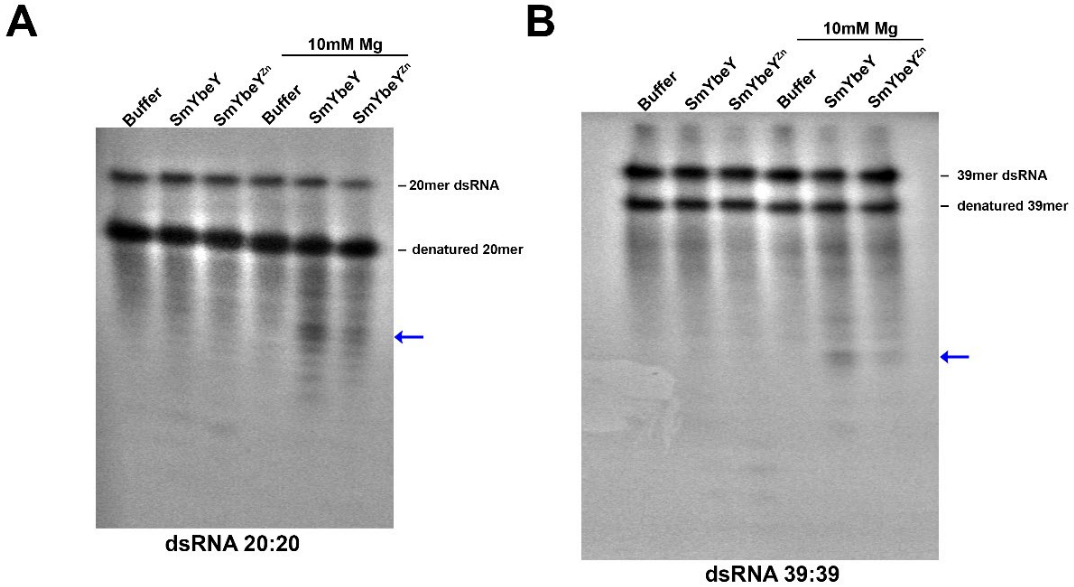

Figure S6. The activity of SmYbeY and SmYbeY^{Zn} on wither 20:20 **(A)** or 39:39 **(B)** double-stranded substrates. Blue arrow indicates the minor degradation products formed in the presence of Mg^{2+}. The indicated substrates are mixed with 25μM of indicated proteins in the presence or absence of 10mM Mg^{2+} and incubated at 37°C for 2 hrs. Panels are representative of at least three independent experiments.

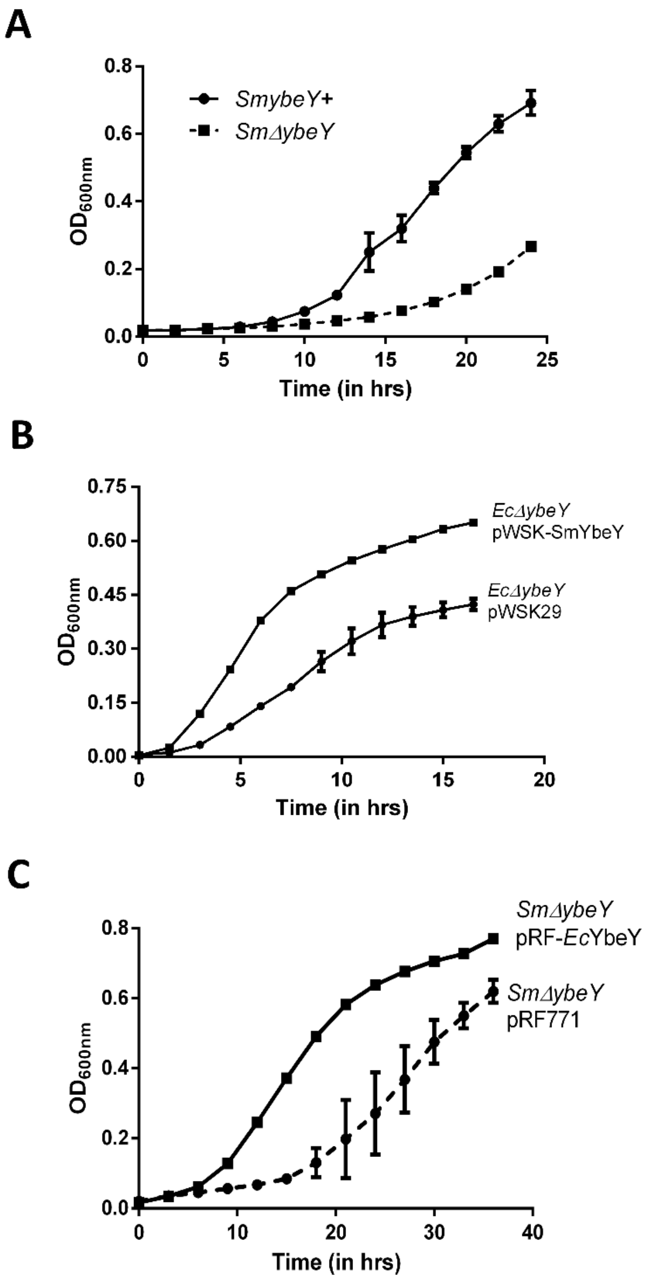

Figure S7. (A) Growth Curves of *S. meliloti* strains with and without YbeY. Cells were grown in LBMC media at 30°C and monitored by measurement of OD_{600nm} for the time indicated. (**B)** Growth of the *EcΔybeY* strains, complemented with empty vector or SmYbeY expressing plasmid, were measured similar to panel A but at 37°C. (**C)** Growth Curves of the *SmΔybeY* strains complemented with empty vector or EcYbeY expressing plasmid were measured as described in panel A.

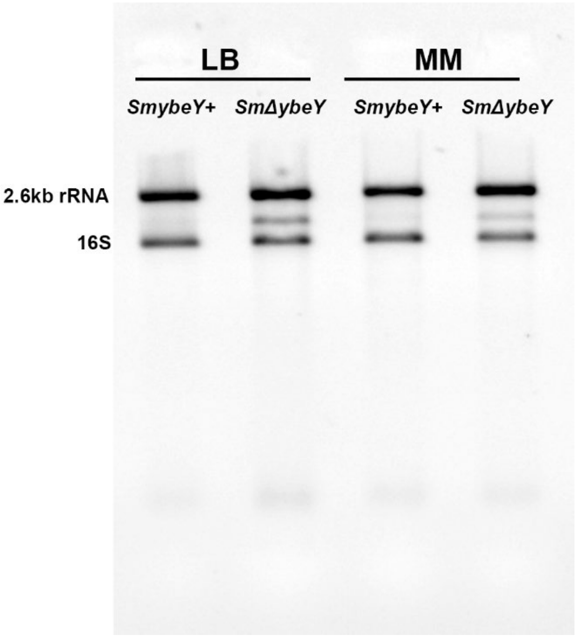

Figure S8. Total RNA was extracted from the SM2B3001 strains with or without YbeY, grown in either the LB media supplemented with Ca and Mg or the minimal media (MM) at 30°C. RNA samples were then subjected to agarose/Synergel electrophoresis. Presence of the unknown band was detected in the SM2B3001 strain lacking YbeY in both the media growth conditions.

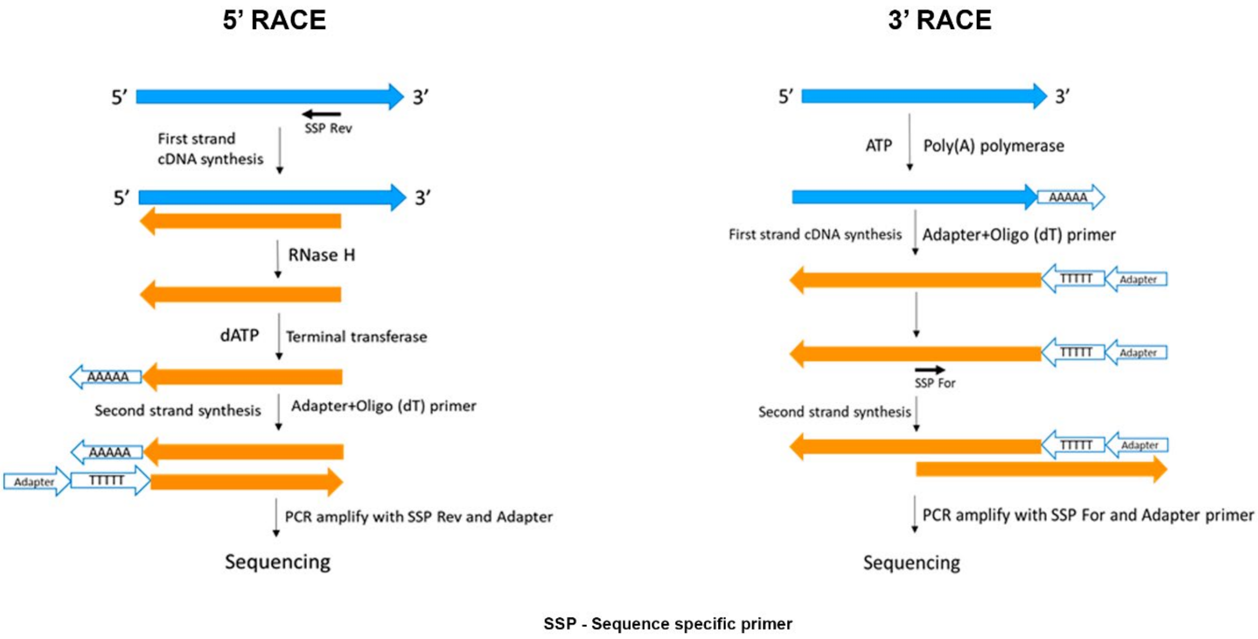

Figure S9. Steps involved in the RACE protocol used to identify the 5' and 3' end of the pre-16S rRNA.
